# Identification of the enzymes responsible for m^2,2^G and acp^3^U formation on cytosolic tRNA from insects and plants

**DOI:** 10.1371/journal.pone.0242737

**Published:** 2020-11-30

**Authors:** Holly M. Funk, Ruoxia Zhao, Maggie Thomas, Sarah M. Spigelmyer, Nichlas J. Sebree, Regan O. Bales, Jamison B. Burchett, Justen B. Mamaril, Patrick A. Limbach, Michael P. Guy

**Affiliations:** 1 Department of Chemistry and Biochemistry, Northern Kentucky University, Highland Heights, Kentucky, United States of America; 2 Rieveschl Laboratories for Mass Spectrometry, Department of Chemistry, University of Cincinnati, Cincinnati, Ohio, United States of America; Universität Stuttgart, GERMANY

## Abstract

Posttranscriptional modification of tRNA is critical for efficient protein translation and proper cell growth, and defects in tRNA modifications are often associated with human disease. Although most of the enzymes required for eukaryotic tRNA modifications are known, many of these enzymes have not been identified and characterized in several model multicellular eukaryotes. Here, we present two related approaches to identify the genes required for tRNA modifications in multicellular organisms using primer extension assays with fluorescent oligonucleotides. To demonstrate the utility of these approaches we first use expression of exogenous genes in yeast to experimentally identify two *TRM1* orthologs capable of forming *N2*,*N2*-dimethylguanosine (m^2,2^G) on residue 26 of cytosolic tRNA in the model plant *Arabidopsis thaliana*. We also show that a predicted catalytic aspartate residue is required for function in each of the proteins. We next use RNA interference in cultured *Drosophila melanogaster* cells to identify the gene required for m^2,2^G_26_ formation on cytosolic tRNA. Additionally, using these approaches we experimentally identify *D*. *melanogaster* gene *CG10050* as the corresponding ortholog of human *DTWD2*, which encodes the protein required for formation of 3-amino-3-propylcarboxyuridine (acp^3^U) on residue 20a of cytosolic tRNA. We further show that *A*. *thaliana* gene *AT2G41750* can form acp^3^U_20b_ on an *A*. *thaliana* tRNA expressed in yeast cells, and that the aspartate and tryptophan residues in the DXTW motif of this protein are required for modification activity. These results demonstrate that these approaches can be used to study tRNA modification enzymes.

## Introduction

Posttranscriptional modification of tRNA is required for efficient and accurate protein translation, and tRNA from all organisms is extensively modified [1]. In the model yeast *Saccharomyces cerevisiae*, defects in certain tRNA modifications can lead to temperature sensitivity, impaired cell growth, or even lethality [2]. Mutations in genes encoding enzymes responsible for tRNA modifications in humans, with their corresponding loss of modifications, cause diseases including intellectual disability [3], mitochondrial disorders [4–8], and familial disautonomia [9–11]. Additionally, genes encoding tRNA modification enzymes have been associated with diseases such as cancer and metabolic disorders [12, 13]. tRNA modifications have also been shown to be involved in response to stress and other environmental stimuli [14–17]. The study of tRNA modifications in multicellular model organisms such as the plant *Arabidopsis thaliana* and the insect *Drosophila melanogaster* has also recently added insight into the roles of modifications in development and disease [18–23]. Due to the roles of tRNA modifications in varied processes, identifying and characterizing the enzymes that form them is of importance in our understanding of gene expression and human health.

The roles that many post-transcriptional modifications play in tRNA function and stability have been identified [24, 25]. In general, modifications in and around the anticodon loop are critical for translational fidelity by ensuring proper tRNA charging [26–28], reading frame maintenance [29–31], and decoding of the RNA [32]. Modifications in the body of the tRNA are generally important for proper folding of the tRNA and for tRNA stability [33–36]. For example, eukaryotic tRNAs lacking certain body modifications undergo degradation by the 5’ to 3’ exonucleases Rat1 and Xrn1 through a process known as rapid tRNA decay (RTD) [33, 37]. This process is exacerbated by high temperature, primarily due to instability of the acceptor and T-stems [38–40]. Some of these modifications in the body of the tRNA enhance stability by preventing unwanted intramolecular base-paring and/or by modulating base-pairing. Thus, the *N2*,*N2*-dimethylguanosine (m^2,2^G) modification found at position 26 of most G26:A44 base-pair containing eukaryotic tRNAs likely modulates non-canonical pairing with A44, and blocks alternative tRNA conformations by blocking base-pairing of G_26_ with other residues [41–44]. The m^2,2^G_26_ modification is formed on both cytosolic and mitochondrial tRNA by Trm1 in yeast [45, 46] and by its homolog TRMT1 in humans [47, 48]. Yeast *TRM1* and human *TRMT1* are encoded in the nucleus, but once expressed, localize to both the nucleus and the mitochondria [47, 49]. Defects in m^2,2^G_26_ cause temperature sensitivity in yeast [50], and intellectual disability in humans [51–54]. Additionally, correct pre-tRNA folding in the yeast *Schizosaccharomyces pombe* was shown to require m^2,2^G_26_ modification by Trm1, and/or La RNA chaperone activity [55].

Most eukaryotic tRNA modifications and the enzymes that form them have been identified experimentally, and both appear to be conserved throughout eukaryotes [1]. For example, of the 25 modifications known to occur on *Saccharomyces cerevisiae* cytoplasmic tRNA, 19 have been found on human cytoplasmic tRNA [1, 56]. To date, genes encoding the majority of the proteins required for all 25 of these modifications in *S*. *cerevisiae* have been identified [2]. Most of these genes were identified using one of two general approaches. In the first approach, yeast homologs were predicted by sequence homology to known tRNA modification enzymes, often from *Escherichia coli*, followed by modification analysis of tRNA from yeast knockout strains. This method was used to identify most of the yeast pseudouridine synthases [57–61], which convert uridine to pseudouridine on yeast tRNA, as well as most of the tRNA methyltransferases [62–68]. In the second approach, several other tRNA modification enzymes were discovered using proteomic libraries to detect in vitro enzymatic activity towards a tRNA substrate [69]. Yeast members of the tRNA dihydrouridine synthase family [70, 71], and three other tRNA methyltransferases [72–74] were discovered using this approach. After their discovery in yeast, many human tRNA modification enzymes have been identified in humans by sequence homology and direct experimental analysis of cells deficient for candidate genes [75].

Humans and other multicellular eukaryotes also contain tRNA modifications that are not found in yeast. For example, some plant and animal tRNAs contain the 3-amino-3-propylcarboxyuridine (acp^3^U) at residues 20, 20a, or 20b on cytoplasmic tRNAs [1, 76]. For a description of tRNA numbering, please see reference [77]. The human enzymes responsible for acp^3^U_20_ and acp^3^U_20a_ were recently identified as DTWD1 and DTWD2, respectively [76]. Lack of these modifications in tandem resulted in slow growth of cultured human cells, suggesting a role for these modifications in translation [76]. This modification is also found at residue 47 of bacterial, mitochondrial, and plastid tRNAs [1]. In bacteria, this modification increases thermal stability [76]. The *E*. *coli* protein YfiP (now named TuaA or TapT) was recently identified as the enzyme that forms acp^3^U_47_ on bacterial tRNA [76, 78], and lack of this modification results in a motility defect and genome instability in cells [76].

**Fig 1:**
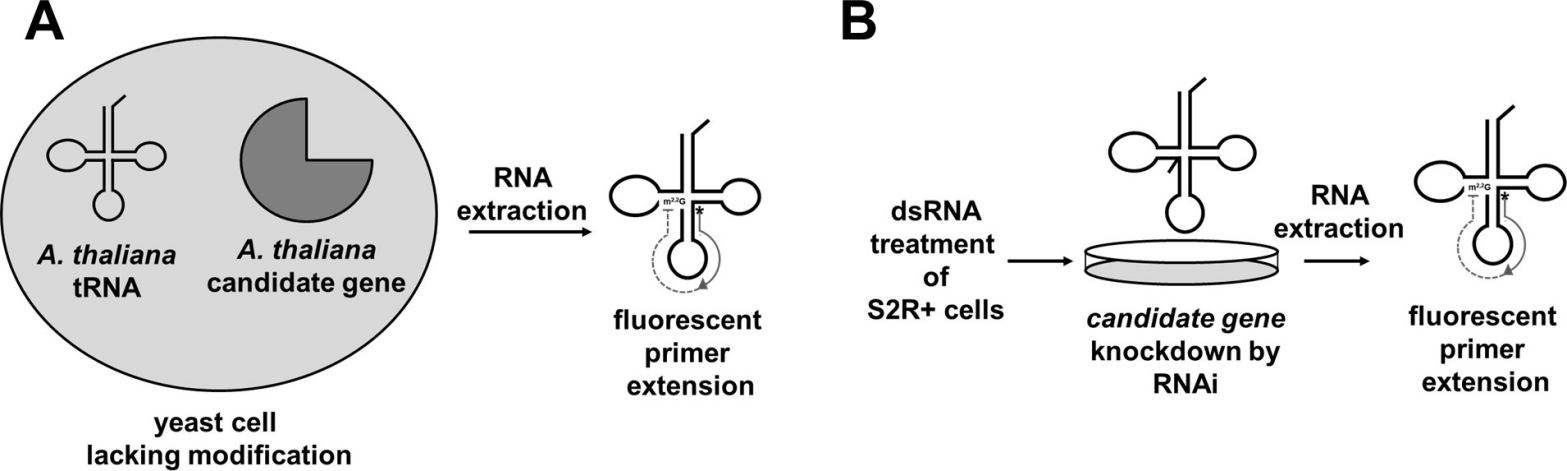
Schematic of approaches to identify tRNA modification enzymes. **(A)** Expression of tRNA and candidate genes in *S*. *cerevisiae*. An exogenous tRNA known or expected to receive the modification of interest, and a candidate modification gene are co-expressed in a yeast strain lacking the modification. RNA is extracted from the yeast and analyzed by primer extension with a fluorescent oligonucleotide specific for the tRNA to determine if the modification is present. **(B)** Analysis of *D*. *melanogaster* tRNA in dsRNA-treated S2R+ cells. S2R+ cells are treated with dsRNA complementary to the gene of interest, and tRNA is analyzed using fluorescent primer extension to detect the modification.

In this report, we describe two different approaches that we use to experimentally identify the plant and insect orthologs of the Trm1 family of enzymes required for the m^2,2^G_26_ modification of cytosolic tRNA. The first approach involves co-expression of an exogenous tRNA and a candidate gene in yeast, followed by detection of the modification by fluorescent primer extension (Fig 1A). Using this approach we identify two *A*. *thaliana* genes that encode enzymes capable of forming m^2,2^G_26_ on cytosolic plant tRNA. In the second approach, we use RNA interference (RNAi) and primer extension analysis to identify the m^2,2^G_26_ enzyme in the model insect *D*. *melanogaster* (Fig 1B). We then use these approaches to identify orthologs of the newly discovered DTWD2 family of proteins that are required for the acp^3^U_20a_ tRNA modification of cytosolic tRNA in *D*. *melanogaster*, and that can form the acp^3^U_20b_ modification on an *A*. *thaliana* tRNA.

## Materials and methods

### Yeast strains and plasmids

Yeast strains used in this study are listed in Table 1. The BY4742 *trm1Δ*::*kanMX* strain was purchased from Dharmacon. The BY4742 *trm1Δ*::*kanMX met22Δ*∷*ble*^*R*^ strain (yMG2129-4) was constructed by standard methods [37]. Plasmids used in this study are listed in Table 2. The *A*. *thaliana TRM1a* (*AT3G02320*), *TRM1b* (*AT5G15810*), *TRM1c* (*AT3G56330*), *DTWD2A* (*AT2G41750*), and *DTWD2B* (*AT5G54880*) yeast expression plasmids were constructed by PCR amplification of genes from an *A*. *thaliana* cDNA library [79] with primers that added PacI and EagI cut sites, followed by digestion of the product with PacI and EagI and ligation into vector BG2596 [80]. Variants of *A*. *thaliana* genes were generated by performing site-directed mutagenesis on plasmids containing wild type genes using the Q5 site-directed mutagenesis kit (Promega) following manufacturer’s instructions. The *A*. *thaliana* tRNA^Leu(AAG)^-G37C plasmid was generated by PCR amplification of the tRNA gene from *A*. *thaliana* genomic DNA with primers that added XhoI and BglII sites, followed by digestion of the product with XhoI and BglII, and ligation into plasmid pMG24A [56] to replace the *S*. *cerevisiae* tRNA^Leu(UAA)^ gene, yielding plasmid pMG669B. A G37C mutation was then added by performing site-directed mutagenesis on plasmid pMG669B using Q5 site-directed mutagenesis to yield *A*. *thaliana* tRNA^Leu(AAG)^-G37C (pMG671B).

**Table 1:** Yeast strains used in this study.

Strain	Genotype	Source
BY4742	*MAT*α *his3-Δ1 leu2Δ0 met15-Δ0 ura3-Δ0*	Open Biosystems
BY4742 *trm1Δ*	BY4742, *trm1Δ*::*kanMX*	Dharmacon
yMG2129-4	BY4742, *trm1Δ*::*kanMX*, *mett22Δ*::*ble*^*R*^	This study

**Table 2:** Plasmids used in this study.

Plasmid	Parent	Description	Source
pBG2596		2μ *URA3* P_*GAL110*_ LIC	(Quartley et al. 2009)
pMG662B	pBG2596	2μ *URA3* P_*GAL10*_ *A*. *thaliana TRM1a*	This study
pMG729E	pBG2596	2μ *URA3* P_*GAL10*_ *A*. *thaliana TRM1a-D200A*	This study
pMG717A	pBG2596	2μ *URA3* P_*GAL10*_ *A*. *thaliana TRM1b*	This study
pMG730E	pBG2596	2μ *URA3* P_*GAL10*_ *A*. *thaliana TRM1b-D200A*	This study
pMG718A	pBG2596	2μ *URA3* P_*GAL10*_ *A*. *thaliana TRM1c*	This study
pMG735B	pBG2596	2μ *URA3* P_*GAL10*_ *A*. *thaliana TRM1c-D172A*	This study
pMG24a		2μ *LEU2 S*. *cerevisiae* tRNA^Leu(UAA)^	(Guy et al. 2012)
pMG669B	pMG24a	2μ *LEU2 A*. *thaliana* tRNA^Leu(AAG)^	This study
pMG671B	pMG669B	2μ *LEU2 A*. *thaliana* tRNA^Leu(AAG)^-G37C	This study
pMG725A	pBG2596	2μ *URA3* P_*GAL10*_ *A*. *thaliana DTWD2A*	This study
pMG726A	pBG2596	2μ *URA3* P_*GAL10*_ *A*. *thaliana DTWD2B*	This study
pMG731C	pBG2596	2μ *URA3* P_*GAL10*_ *A*. *thaliana DTWD2A-D133A*	This study
pMG732E	pBG2596	2μ *URA3* P_*GAL10*_ *A*. *thaliana DTWD2A-W136A*	This study

### RNA isolation

Yeast strains were grown in YPD or selective synthetic media to late log phase. For primer extension, qRT-PCR, and tRNA purification, bulk low molecular weight RNA was extracted using hot phenol as previously described [72]. RNA extraction from *D*. *melanogaster* S2R+ cells was performed using TRIzol (Thermo Fisher) according to manufacturer’s instructions.

### Primer extension assays

For primer extension, 10–20 μmol 5’ Tye665-labeled oligonucleotides (Integrated DNA Technologies) was annealed to 2–10 μg RNA, heated to 95°C and slow cooled to 37°C. Labeled oligonucleotides are listed in Table 3. The entire reaction was then incubated with 1 mM dNTPs and 1.89 U of Avian Myeloblastosis Virus (AMV) reverse transcriptase. Reactions were incubated at 37°C overnight, and analyzed by 15% PAGE with 7M Urea. The gel was placed between overhead projector sheets and visualized using a Typhoon 9200 scanner with a 620 BP30 Cy5 emission filter at high sensitivity and PMT between 600–800. Sequencing reactions were annealed similarly and extended overnight with the addition of 0.1mM ddNTP’s. Primer MPG1772 was used for detection of m^2,2^G_26_ on *A*. *thaliana* tRNA^Leu(AAG)^-G37C, and MPG1587 was used for detection of acp^3^U_20b_ on the same tRNA.

**Table 3:** Tye665-labeled oligonucleotides used for primer extension.

Primer	Target tRNA	Sequence	Target Nucleotides
MPG951	*S*. *cerevisiae* tRNA^Tyr^	CGAACGCCCGATCTCAAGATT	55–37
MPG1012	*S*. *cerevisiae* tRNA^Trp^	GTGAAACGGACAGGAATTGAACCTG	74–50
MPG1587	*A*. *thaliana* tRNA^Leu(AAG)^-G37C	CTTTCGGACCAGAAGCTTAATCT	e5-29
MPG1772	*A*. *thaliana* tRNA^Leu(AAG)^-G37C	CCTTTCGGACCAGAAGCTTAA	47–33
MPG1045	*D*. *melanogaster* tRNA^Tyr^	GAACCAGCGACCTATGGATCTACAG	56–32
MPG1126	*D*. *melanogaster* tRNA^Val(CAC)^	GAACCGGGGACCTTGTGCGTGTG	56–34

### Northern blot analysis

Bulk RNA was analyzed by PAGE and transferred to a nitrocellulose membrane as previously described [33]. For detection by Northern blot, *S*. *cerevisiae* tRNA^Trp^ was detected with 5’ Tye665-labeled probe MPG1012 and *A*. *thaliana* tRNA^Leu(AAG)^-G37C was detected with MPG1587 (Table 3).

### Quantitative Real Time PCR (qRT-PCR)

RNA was treated with RQ1 RNase-Free DNase (Promega) and then reverse transcribed using a Verso cDNA Kit (Thermo Scientific) using a 3:1 (v/v) mix of random hexamers and anchored oligo-dT primers. DNA was then PCR amplified using DyNAmo HS SYBR™ Green qPCR Kit (Thermo Scientific) master mix and primers specific to indicated genes. Primers used for qRT-PCR are listed in Table 4. In yeast, RNA was normalized to *ACT1*, and in *D*. *melanogaster*, RNA was normalized to *Act42A*.

**Table 4:** Oligonucleotides used for qRT-PCR.

Primer	Organism	Target	Sequence
MPG1598	*S*. *cerevisiae*	*ACT1*	GAAATGCAAACCGCTGCTCA
MPG1599	*S*. *cerevisiae*	*ACT1*	TACCGGCAGATTCCAAACCC
MPG1756	*S*. *cerevisiae*	*TUB1*	CCAAGGGCTATTTACGTGGA
MPG1757	*S*. *cerevisiae*	*TUB1*	GGTGTAATGGCCTCTTGCAT
MPG1617	*A*. *thaliana*	*TRM1a*	GGGACTCATGTGAATCCGCT
MPG1618	*A*. *thaliana*	*TRM1a*	CGAACCAATGTGACGCGAAA
MPG1652	*A*. *thaliana*	*TRM1b*	CCACGTCGGTTCGCTTAGTA
MPG1653	*A*. *thaliana*	*TRM1b*	CGACCTGCCCTTATCTTGGG
MPG1654	*A*. *thaliana*	*TRM1c*	AATGAGATTGGGCTGCGGAT
MPG1655	*A*. *thaliana*	*TRM1c*	CATGTAGCTTCCCACGGTGA
MPG1266	*D*. *melanogaster*	*TRM1*	CGGAAGAAGGTCAAGGAACA
MPG1267	*D*. *melanogaster*	*TRM1*	GATCCGAAGTCCATCCTCATATC
MPG1635	*D*. *melanogaster*	*ACT42a*	CAAGAGTACGACGAGTCGGG
MPG1636	*D*. *melanogaster*	*ACT42a*	TTCGATGAGGAACGACCACG
MPG1706	*D*. *melanogaster*	*DTWD2A*	GTCCGTCATCAAACCGGACT
MPG1707	*D*. *melanogaster*	*DTWD2A*	GCATGGTAGGTGTGCTTTGC
MPG1708	*A*. *thaliana*	*DTWD2A*	TGGTCAAAGCTAGCGAGGTG
MPG1709	*A*. *thaliana*	*DTWD2A*	CTCCACTCACTGATGCGTCT
MPG1710	*A*. *thaliana*	*DTWD2B*	CAAAGAAGTACGACGGCAGC
MPG1711	*A*. *thaliana*	*DTWD2B*	TTCACCGATCTCCTTCATCGC

### tRNA purification

tRNA^Leu(AAG)^-G37C was purified from yeast total RNA extracts using a 5’-biotinylated oligonucleotide with the sequence 5’-TGGTGTTGACAGTGGGATTTGAACCC-3’, which is complementary to nucleotides 76–51 in the tRNA, as previously described (72). Briefly, biotinylated oligonucleotide bound to streptavidin magnetic particles was incubated with yeast RNA to capture tRNA^Leu(AAG)^-G37C. Bound tRNA was then washed extensively to remove non-specific tRNA. Eluted tRNA was quantified using A_260_ prior to LC-MS/MS analysis.

### LC-MS/MS analysis of nucleosides

tRNA (2–4 μg) was digested to single nucleosides as previously described using nuclease P1, snake venom phosphodiesterase, and bacterial alkaline phosphatase [81, 82]. The lyophilized samples were reconstituted in mobile phase A. Reversed phase chromatography was carried out with a high-strength silica column (Acquity UPLC HSS TS, 1.8 μm, 1.0 mm X 100 mm, Waters, Milford, MA) on an ultra-high-performance liquid chromatography (UHPLC) system (Vanquish Flex Quaternary, Thermo Fisher Scientific, San Jose, CA). Mobile phase A contained 5.3 mM ammonium acetate in water, pH 5.3, and mobile phase B contained a mixture of acetonitrile/water (40:60) with 5.3 mM ammonium acetate.

The gradient program consisted of: 0% B (from 0 to 7.6 min), 2% B at 15.7 min, 3% B at 19.2 min, 5% B at 25.7 min, 25% B at 29.5 min, 50% B at 32.3 min, 75% B at 36.4 min (hold for 0.2 min), 99% B at 39.6 min (hold for 7.2 min), then returning to 0% B at 46.9 min. After that, a re-equilibration step at 0% B for 18.1 min was employed prior to the next injection. A flow rate of 100 μL min^-1^ was used. The column temperature was set at 30°C.

An Orbitrap Fusion^TM^ Lumos^TM^ Tribrid mass spectrometer (Thermo Fisher Scientific) interfaced with a heated electrospray (H-ESI) source was used for the identification and relative quantification of m^2,2^G. All instrument parameters were as described previously [83], except that the collision-induced dissociation (CID) energy was set at 40%, and higher-energy collisional dissociation (HCD) energy was set at 80 arbitrary units.

Quantiva^TM^ triple quadrupole mass spectrometer interfaced with an H-ESI source (Thermo Fisher Scientific) was used for the identification and relative quantification of acp^3^U. Instrument settings included detection in positive polarity with an H-ESI electrospray voltage of 3.8 kV, Ion Transfer Tube temperature at 290°C, vaporizer temperature of 100°C, sheath gas, auxiliary gas and sweep gas at 35, 10, 0 arbitrary units respectively. Collision gas pressure was 1.5 mTorr. Q1 and Q3 Resolution (FWHM) was set at 0.7 Da. Selected reaction monitoring (SRM) transitions for acp^3^U were m/z 346–214, 346–197, 346–168 with collision energy (CE) of 20 V, RF lens was 58 V. SRM transition for U was m/z 245–113 with collision energy (CE) of 10 V and RF lens 30 V.

Data were analyzed using Qual browser of Xcalibur 3.0.

### dsRNA generation

dsRNA for RNAi was generated by PCR amplification of the desired gene from *D*. *melanogaster* genomic DNA with primers containing 5’ T7 promoter sites. Primers used for dsRNA generation are listed in Table 5. Regions for dsRNA were selected using the *Drosophila* RNAi Screening Center (DRSC) website to minimize off-target effects. The PCR product was analyzed by agarose gel, purified using a QIAquick gel extraction kit under low salt conditions (Qiagen), and 0.2 μg was used as a template for in vitro transcription using a T7 Megascript kit and purified according to manufacturer’s instructions (Thermo Fisher).

**Table 5:** Oligonucleotides used for D. *melanogaster* dsRNA generation.

Primer	Target	Sequence
MPG932	*TRM1*	GAATTAATACGACTCACTATAGGGAGATCTTTCCATGGTCCAAGAGG
MPG933	*TRM1*	GAATTAATACGACTCACTATAGGGAGAGATAATGCCGATTTGCGACT
MPG736	*TRM7*	GAATTAATACGACTCACTATAGGGAGAGAGTGCCTTCAAGTTGCTCC
MPG737	*TRM7*	GAATTAATACGACTCACTATAGGGAGAGCATCTGGGAGCTCAATAGC
MPG1698	*DTWD2A*	GAATTAATACGACTCACTATAGGGAGAACACCCTGCAGAGGAGAAGA
MPG1699	*DTWD2A*	GAATTAATACGACTCACTATAGGGAGAAGTCACTTATACCCACGGCG
MPG1700	*DTWD2A*	GAATTAATACGACTCACTATAGGGAGATCTACAAAGGCAAGCGGTTT
MPG1701	*DTWD2A*	GAATTAATACGACTCACTATAGGGAGAAGGCGGTGTTTCTAAGCAGA

### *D*. *melanogaster* S2R+ cell culture and RNAi treatment

*D*. *melanogaster* S2R+ cells [84] were grown in Schneider’s medium (Gibco) + 10% fetal bovine serum (FBS) (Gibco) at 27°C in culture flasks. RNAi was performed in 6-well plates as described on the DRSC website, essentially as previously described [85]. Briefly, 2 X 10^6^ cells were plated to each well of a 6-well plate in 1 mL of serum free media, 10 μg of dsRNA was added to each well, and cells were incubated at room temperature for 30 minutes. After incubation, 3 mL of media + 10% FBS was added to each well. Cells were harvested after 3 days, or for multiple RNAi treatments, medium was removed, each well was resuspended in 1 mL of serum-free medium, and 250 μL of cells were plated in new wells already containing 750 μL serum-free medium. Cells were then treated with dsRNA as previously described and harvested or passaged again after 3 days.

## Results and discussion

### Primer extension assays using fluorescent oligonucleotides sensitively detect m^2,2^G_26_ on tRNA from yeast cells

**Fig 2:**
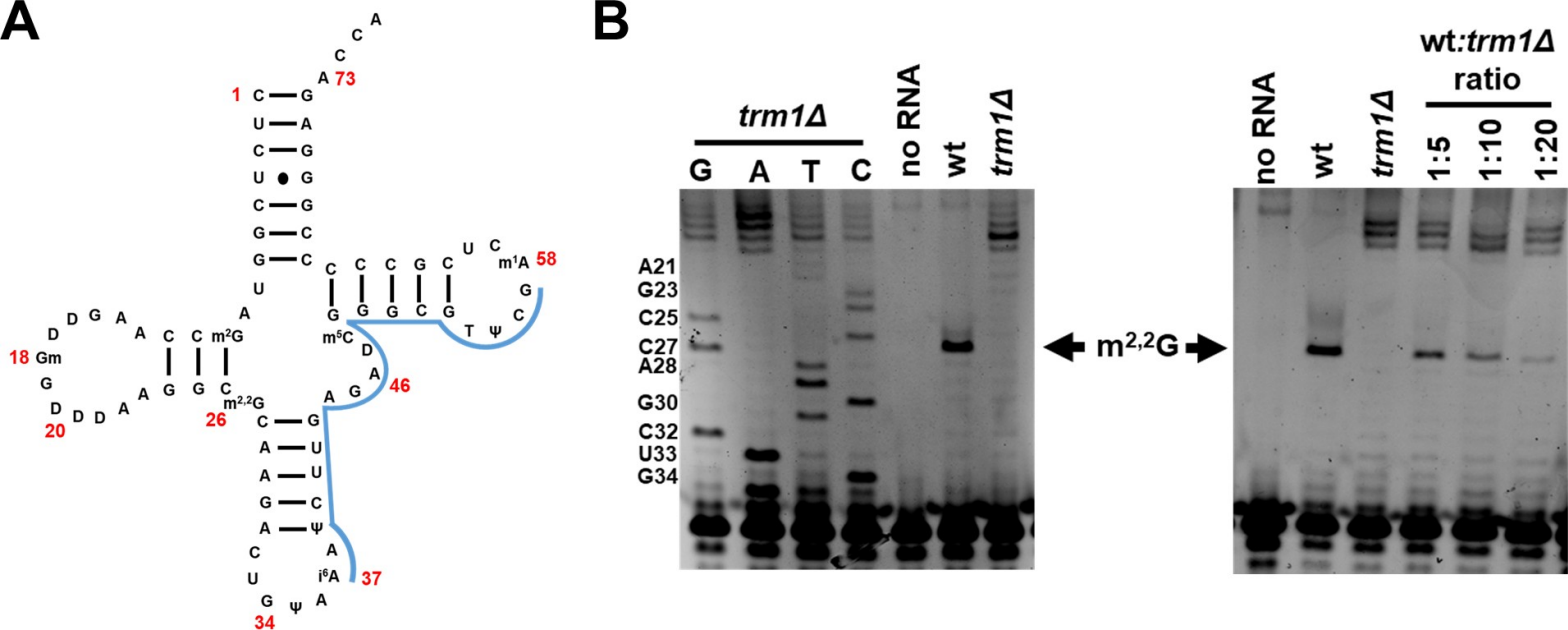
Detection of m^2,2^G_26_ by fluorescent primer extension in yeast cells. **(A)** Schematic of *S*. *cerevisiae* tRNA^Tyr^. Location of primer binding is shown in blue. Selected nucleotides are numbered in red. **(B)** Detection of m^2,2^G_26_ by fluorescent primer extension in yeast cells. Left, bulk RNA was extracted from indicated strains and analyzed by primer extension to yeast tRNA^Tyr^. Selected nucleotides from the sequencing reactions are provided on the left of the gel. Right, bulk RNA was extracted from culture of indicated individual strains or culture from mixtures of strains in indicated ratios, and then analyzed by primer extension to yeast tRNA^Tyr^.

We first determined whether fluorescently-labeled oligonucleotides could be used to sensitively detect tRNA modifications in yeast by primer extension [78, 86]. Thus, we used a Tye665 5’-end labeled oligonucleotide specific to *Saccharomyces cerevisiae* tRNA^Tyr^ (Fig 2A) to perform primer extension on RNA from wild type and *trm1Δ* cells, to detect the m^2,2^G_26_ modification. In a primer extension assay, the presence of a base-pair blocking modification such as m^2,2^G prohibits incorporation of a nucleotide complementary to the modified nucleotide by reverse transcriptase. Thus, presence of m^2,2^G_26_ results in a stop at residue 27 of the tRNA when analyzed by polyacrylamide gel electrophoresis (PAGE). We mixed wild type cells and *trm1Δ* cells in different ratios, extracted small molecular weight RNA, and then performed a primer extension assay specific to tRNA^Tyr^. PAGE of the reactions showed a robust block at position 27 on tRNA^Tyr^ from wild type cells (Fig 2B), whereas a block at this position was not observed on tRNA^Tyr^ from *trm1Δ* cells. Instead, a larger band representing a primer extension block closer to the 5’ end of tRNA^Tyr^ was observed, which corresponds to the string of dihydrouridines found in the D-loop of the tRNA (Fig 2A and 2B). Detection of m^2,2^G by this method appears to be sensitive, because a primer extension block corresponding to m^2,2^G_26_ could be easily detected in tRNA from wild-type cells mixed with *trm1Δ* cells at a ratio of 1/10, with detection at a ratio of 1/20 also possible (Fig 2B). Our ability to detect the m^2,2^G_26_ modification in wild-type cells from a background of *trm1Δ* cells indicates that this technique is sensitive enough to detect cells with a tRNA modification from a pool of cells lacking the modifications, which could be useful in the development of a screen to identify genes required for tRNA modifications. This sensitivity also further suggests that in many instances fluorescence could be a viable alternative to traditional primer extension [78, 86], which uses ^32^P. ^32^P has exquisite sensitivity, and is critical for the study of tRNA modifications. However, the use of radioactivity has several drawbacks, which include expense, safety concerns requiring extensive additional training of personnel, and a relatively short window in which the radioactivity can be used, due to the short half-life of ^32^P [86].

### Identification of *A*. *thaliana* TRM1 enzymes using yeast cells and primer extension

We next sought to identify tRNA modification genes from multicellular organisms by expressing the genes in yeast and using fluorescence-based primer extension to detect modification of the tRNA. This approach could provide some advantage over purely in vitro assays, because subcellular trafficking and localization of the protein in yeast could yield insights into the tRNA specificity of the enzyme. However, it is also possible that exogenously expressed proteins could be targeted incorrectly in yeast, thus confounding results. Nonetheless, this system provides a convenient method to rapidly determine if an enzyme has the ability to modify a given tRNA. To demonstrate the feasibility of this approach, we determined which predicted TRM1 enzymes can form the m^2,2^G_26_ modification on *Arabidopsis thaliana* tRNA. Interest in the study of tRNA modifications in *A*. *thaliana* has increased, with recent reports showing that tRNA modifications are involved in development [20, 87–89], disease resistance [18, 90], and stress response [23, 91, 92].

**Fig 3:**
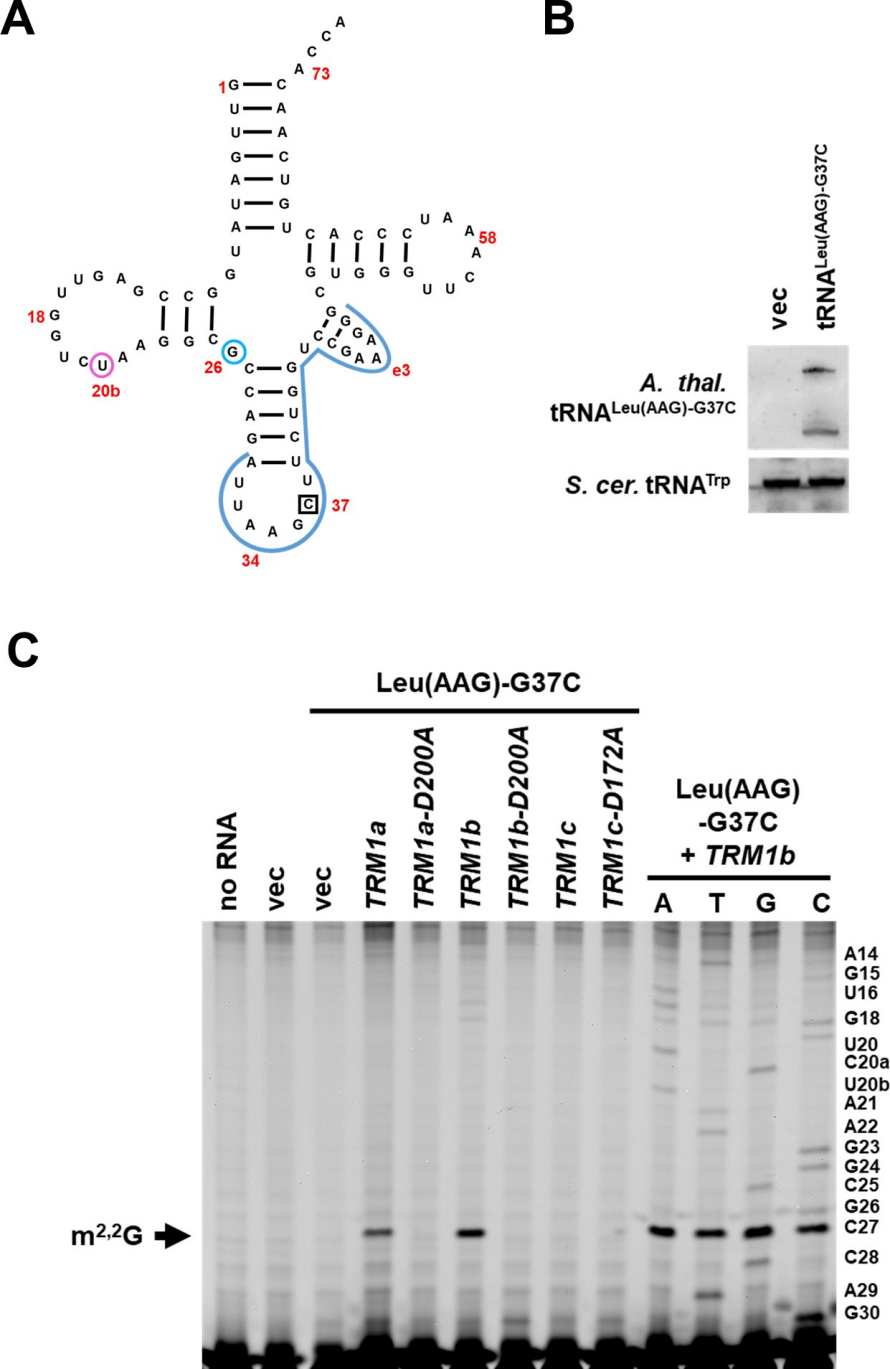
Identification of *TRM1* genes from *A*. *thaliana* in yeast cells. **(A)** Schematic of *A*. *thaliana* tRNA^Leu(AAG)^-G37C. Location of primer binding is shown in blue. G37C mutation is boxed. Predicted location of m^2,2^G_26_ (cyan) and acp^3^U_20b_ (magenta) modifications are circled. Selected nucleotides are numbered in red. **(B)** *A*. *thaliana* tRNA^Leu(AAG)^-G37C is expressed in yeast cells. RNA was extracted from yeast expressing the indicated plasmid (vertical label) and analyzed by Northern blot to the indicated probe (horizontal label) as described in Materials and methods. **(C)** TRM1a or TRM1b expression results in a primer extension block consistent with m^2,2^G_26_ on *A*. *thaliana* tRNA. Bulk RNA was extracted from a *trm1Δ* yeast strain expressing the indicated plasmids and then analyzed by primer extension to *A*. *thaliana* tRNA^Leu(AAG)^-G37C.

There are three known m^2^G/m^2,2^G_26_ methyltransferases in eukaryotes, which include Trm1, Trm11, and Trm14 [45, 68, 93]. All three of these eukaryotic enzymes have a similar Rossman-fold methyltransferase domain, but unlike Trm11 and Trm14 proteins, Trm1 proteins lack a THUMP domain which is involved in tRNA binding [94]. Three predicted *TRM1* candidate genes have been identified in *A*. *thaliana* based on sequence homology [23] (S1 Fig), and the lack of a THUMP domain. To determine if these Trm1 proteins form m^2,2^G_26_, we co-expressed an *A*. *thaliana* tRNA with *A*. *thaliana TRM1* candidate genes in *S*. *cerevisiae*, and then detected m^2,2^G_26_ by fluorescent primer extension. To monitor m^2,2^G modification upon expression of *A*. *thaliana* candidate genes, we generated a high copy yeast expression plasmid encoding *A*. *thaliana* tRNA^Leu(AAG)^ with a G37C mutation (tRNA^Leu(AAG)^-G37C). The modification profile of cytosolic *A*. *thaliana* tRNA^Leu(AAG)^ has not been determined, but cytosolic tRNA^Leu(AAG)^ from the plant *Lupinus luteus* contains a m^2,2^G_26_ modification [95], and *A*. *thaliana* tRNA^Leu(AAG)^ has a G at position 26 [1], suggesting that the *A*. *thaliana* tRNA should also receive the m^2,2^G_26_ modification in the presence of a TRM1 enzyme. Moreover, because *L*. *luteus* tRNA^Leu(AAG)^ contains a 1-methylguanosine (m^1^G) modification at residue 37, and *A*. *thaliana* tRNA^Leu(AAG)^ contains a G37 [1], we added a G37C mutation to *A*. *thaliana* tRNA^Leu(AAG)^ to ensure that G37 on the *A*. *thaliana* tRNA would not be modified to m^1^G by yeast Trm5 [62] (Fig 3A). m^1^G blocks base-pairing, and would therefore likely interfere with primer binding or primer extension in the assay.

We transformed yeast *trm1Δ* cells with the plasmid expressing cytosolic *A*. *thaliana* tRNA^Leu(AAG)^-G37C and with a high copy plasmid expressing *A*. *thaliana* gene *TRM1a* (*AT3G02320*) [23] under control of the P_*GAL*_ promoter, and then detected the m^2,2^G_26_ modification on tRNA^Leu(AAG)^-G37C using a primer extension assay. Cells were grown in selective media containing galactose, and were grown at room temperature to decrease the likelihood that the tRNA^Leu(AAG)^-G37C would be degraded by RTD [33, 40]. Northern blot analysis using a fluorescent oligonucleotide specific to *A*. *thaliana* tRNA^Leu(AAG)^-G37C tRNA demonstrated that the tRNA was readily expressed from the plasmid (Fig 3B). Two bands of differing mobilities specific to tRNA^Leu(AAG)^-G37C were detected. Expression of *TRM1a* mRNA in cells transformed with a *TRM1a* expression plasmid was confirmed by quantitative real-time PCR (qRT-PCR) (Table 6). Primer extension specific to *A*. *thaliana* tRNA^Leu(AAG)^-G37C resulted in a primer extension block consistent with the m2,2G_26_ modification in *trm1Δ* cells when *TRM1a* was expressed (Fig 3C), suggesting that TRM1a is a Trm1 ortholog in *A*. *thaliana*.

**Table 6:** Relative mRNA levels of *A*. *thaliana TRM1* homologs expressed in yeast.

A. thaliana gene	target mRNA	relative levels^a^
*TRM1a*	*TRM1a*	1.51 ± 0.34
*TRM1a-D200A*	*TRM1a*	8.76 ± 2.41
*TRM1b*	*TRM1b*	68.6 ± 35.2
*TRM1b-D200A*	*TRM1b*	32.4 ± 9.98
*TRM1c*	*TRM1c*	12.86 ± 5.11

^a^Relative to *S*. *cerevisiae* tubulin (*TUB1*), after both genes were normalized to *S*. *cerevisiae* actin (*ACT1*). Values are from three independent growths.

Because there are two other predicted *TRM1* genes in *A*. *thaliana* [23], we also tested the ability of *TRM1b* (*AT5G15810*) and *TRM1c* (*AT3G56330*) to form m^2,2^G on *A*. *thaliana* tRNA^Leu(AAG)^-G37C from high copy expression plasmids in yeast. Expression of *A*. *thaliana TRM1b* resulted in a primer extension block on *A*. *thaliana* tRNA^Leu(AAG)^-G37C consistent with the presence of m^2,2^G_26_, whereas expression of *A*. *thaliana TRM1c* did not result in a block within the limits of our detection (Fig 3C). The lack of m^2,2^G formation on tRNA from cells expressing *TRM1c* was not due to lack of mRNA expression (Table 6). This data suggests that TRM1b is also a Trm1 ortholog. It is possible that TRM1c is responsible for the m^2,2^G_26_ modification found on several plastid tRNAs [1], because it is predicted to contain a chloroplast transit peptide and is predicted to localize to this cellular organelle [96, 97]. We cannot rule out the possibility that expression of *TRM1c* failed to generate a detectable primer extension block due to lack of protein expression and/or incorrect subcellular localization of the protein resulting from expression of this exogenous plant gene in yeast.

To further determine if the primer extension block observed upon the expression of TRM1b is due to Trm1 activity, we mutated a predicted active site aspartate residue [98] in Trm1a and in TRM1b (S1 Fig), expressed the variants in yeast, and measured modification activity by primer extension assay. Cells expressing a TRM1a or TRM1b variant containing an alanine substitution of the predicted active site aspartate (D200A) failed to generate a primer extension block (Fig 3C), even though the genes were expressed (Table 6). These data further support the conclusion that TRM1a and TRM1b are *A*. *thaliana* Trm1 orthologs.

To verify that the primer extension block observed on *A*. *thaliana* tRNA^Leu(AAG)^-G37C at position 27 was due to the m^2,2^G_26_ modification, we purified tRNA^Leu(AAG)^-G37C in the presence or absence of TRM1a or TRM1b, and analyzed nucleotide content by liquid chromatography followed by mass spectrometry (LC-MS). To this end, tRNA^Leu(AAG)^-G37C was expressed in the presence or absence of *A*. *thaliana TRM1* genes in *trm1Δ mett22Δ* yeast, purified using a biotinylated nucleotide specific to the tRNA, and then digested to nucleosides. The *met22Δ* mutation was included in these cells to inhibit the rapid tRNA decay pathway [37], which would be expected to reduce degradation of tRNA^Leu(AAG)^-G37C and increase tRNA yields. Nucleosides were separated by reversed phase HPLC and identified using a high-resolution, accurate-mass (HRAM) orbitrap mass spectrometer [83]. The m^2,2^G modification on tRNA^Leu(AAG)^-G37C purified from cells expressing TRM1a or TRM1b was readily detected using this method, whereas levels of m^2,2^G on the same tRNA purified from cells expressing a vector were near background (S2 Fig). We therefore conclude that *A*. *thaliana* TRM1a and TRM1b form m^2,2^G on *A*. *thaliana* tRNA^Leu(AAG)^.

Our finding that *A*. *thaliana* has two enzymes capable of forming cytosolic m^2,2^G_26_ is different than what has been observed in yeast and humans, which each have been shown to only encode one enzyme responsible for formation of cytosolic m^2,2^G_26_ [45, 47]. Although humans encode the TRMT1L protein, which is related to TRMT1 by sequence, it does not form m^2,2^G_26_ on cytosolic tRNA [47]. Human TRMT1L and *A*. *thaliana* TRM1c share only 22% sequence identity, whereas human TRMTL1 shares 27% identity with *A*. *thaliana* TRM1a, and *A*. *thaliana* TRM1c shares 27% identity with human TRMT1. This sequence data strongly suggests that TRMTL and TRM1c do not have similar functions. Homologs to *A*. *thaliana* TRM1c are found in a wide array of plant species with amino acid sequence identities ranging from 37% in the green algae *Micromonas pusilla* to 68% in *Carica papaya* (S3 Fig). Furthermore, as mentioned previously, TRM1c contains a predicted chloroplast transit peptide. Thus, it is highly likely that TRM1c is responsible for m^2,2^G_26_ modification of plastid tRNA.

### Identification of the *D*. *melanogaster* TRM1 enzyme in cultured cells using RNA interference and primer extension

**Fig 4:**
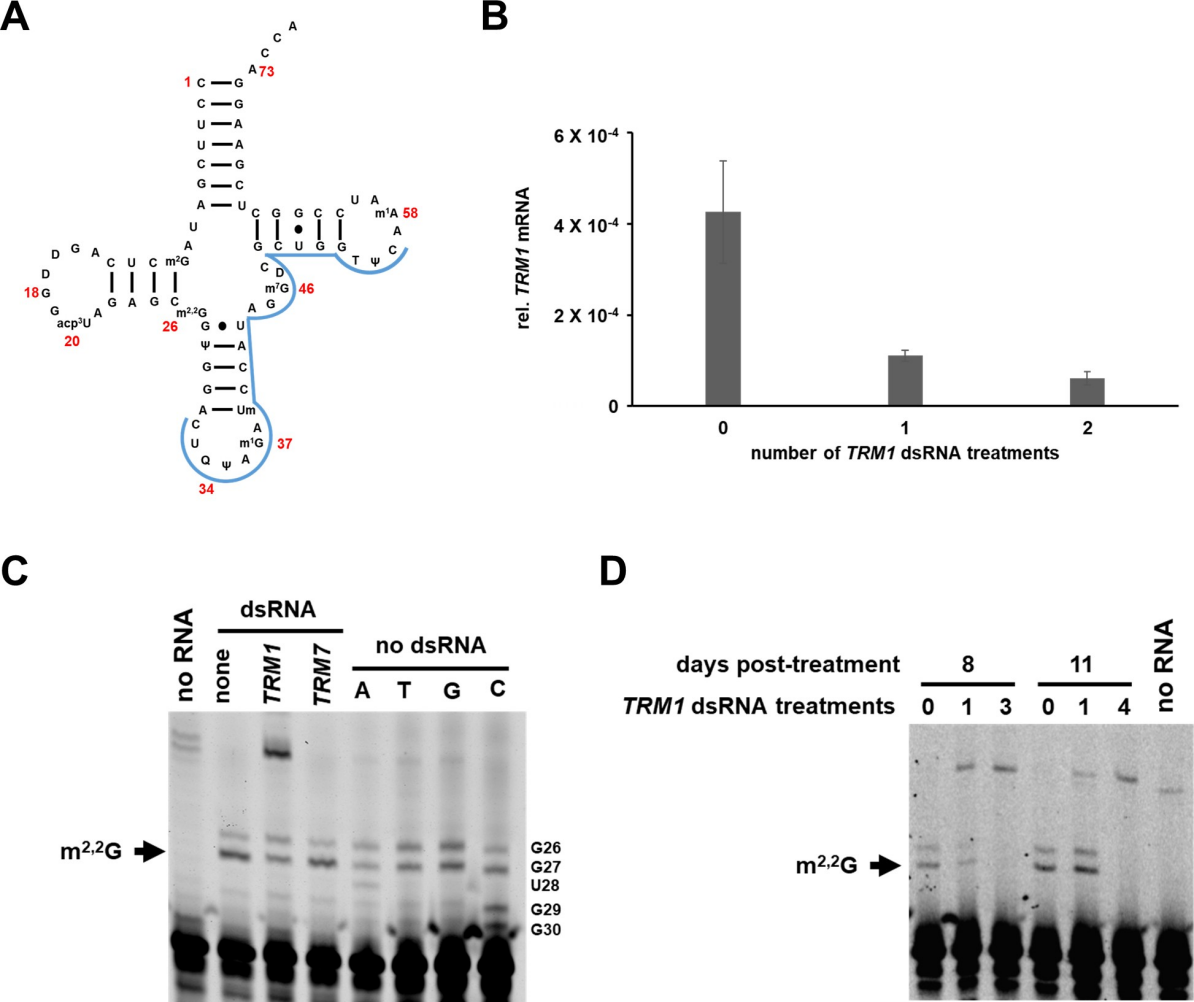
*CG6388* encodes the TRM1 enzyme in *D*. *melanogaster*. **(A)** Schematic of *D*. *melanogaster* tRNA^Tyr^. Location of primer binding is shown in blue. Selected nucleotides are numbered in red. **(B)** *TRM1* mRNA levels significantly decrease in dsRNA-treated S2R+ cells. Cells were treated with dsRNA to *TRM1* as indicated and mRNA levels were measured by qRT-PCR. RNA levels are expressed relative to actin (*ACT42a*). **(C)** Knockdown of *CG6388* by RNAi results in loss of a primer extension block consistent with m^2,2^G_26_. S2R+ cells were treated twice over 6 days with dsRNA to indicated gene as described in Materials and methods. After harvest of cells, RNA was extracted and primer extension to tRNA^Tyr^ was performed. **(D)** Time course of *CG6388* knockdown by RNAi. S2R+ cells were treated as indicated, RNA was extracted, and primer extension to tRNA^Tyr^ was performed.

In a separate approach to identify tRNA modification enzymes in a multicellular eukaryote, we used RNA interference (RNAi) coupled with fluorescent primer extension to identify the *D*. *melanogaster* TRM1 enzyme. Robust RNAi can be achieved by treating cultured S2R+ cells with double stranded RNA (dsRNA) specific to the gene of interest generated directly from a PCR product [85, 99–101]. We tested the effect of RNAi to the predicted *D*. *melanogaster TRM1* gene (*CG6388*), followed by primer extension to detect m^2,2^G on tRNA^Tyr^, which receives the modification (Fig 4A) [102]. We treated S2R+ cells with dsRNA to *TRM1* twice over the course of 6 days, harvested cells, extracted RNA, and used fluorescent primer extension specific to tRNA^Tyr^ to detect the presence of m^2,2^G. Quantification of *TRM1* gene expression at day 6 by qRT-PCR revealed that *TRM1* mRNA levels were significantly reduced compared to untreated cells (Fig 4B), verifying that dsRNA treatment resulted in a significant reduction in gene expression. Primer extension specific to tRNA^Tyr^ from cells treated with no dsRNA or with dsRNA to *D*. *melanogaster TRM7/FTSJ1* [99, 103] resulted in the presence of two primer extension blocks, the darker, bottom one of which was consistent with the size expected for an m^2,2^G modification on residue 26 (Fig 4C). In contrast, primer extension analysis of tRNA^Tyr^ from cells treated with dsRNA to *TRM1* showed a marked decrease in the intensity of the bottom band of the doublet, with a corresponding appearance of a band of lesser mobility, consistent with the presence of the acp^3^U modification found at position 20 of the tRNA [102] (Fig 4C, S4 Fig). No obvious difference in cell growth or morphology was observed between cells treated with either *TRM1* or *TRM7* dsRNA and untreated cells. The top band of the doublet is consistent with a pause at residue C_25_, so although *D*. *melanogaster* tRNA^Tyr^ has G residues at positions 26 and 27, it does not appear that the doublet is due to m^2,2^G modification of both residues 26 and 27, as has been observed for human tRNA^Tyr^ [104] and for tRNA^Cys^ from the eubacterium *Aquifex aeolicus* [105].

To further optimize RNAi for knockdown of tRNA modifications in S2R+ cells, we varied the number of dsRNA treatments over the course of 8 and 11 days, and harvested cells at different time points. We found that three dsRNA treatments of cells over the course of 8 days was sufficient to knock out nearly all detectable levels of the primer extension block corresponding to m^2,2^G (Fig 4D). Thus, our results demonstrate that *CG6388* encodes the TRM1 enzyme responsible for the m^2,2^G_26_ modification in *D*. *melanogaster*, because tRNA^Tyr^ in *D*. *melanogaster* has previously been shown to have an m^2,2^G_26_ modification [102], and because of the high sequence homology between this *D*. *melanogaster* protein and other bona fide Trm1 proteins. These results also suggest that this system could be used to rapidly screen candidate genes for novel tRNA modifications in *D*. *melanogaster*.

### DTWD2 homologs are responsible for the acp^3^U_20a_ modification in flies and plants

**Fig 5:**
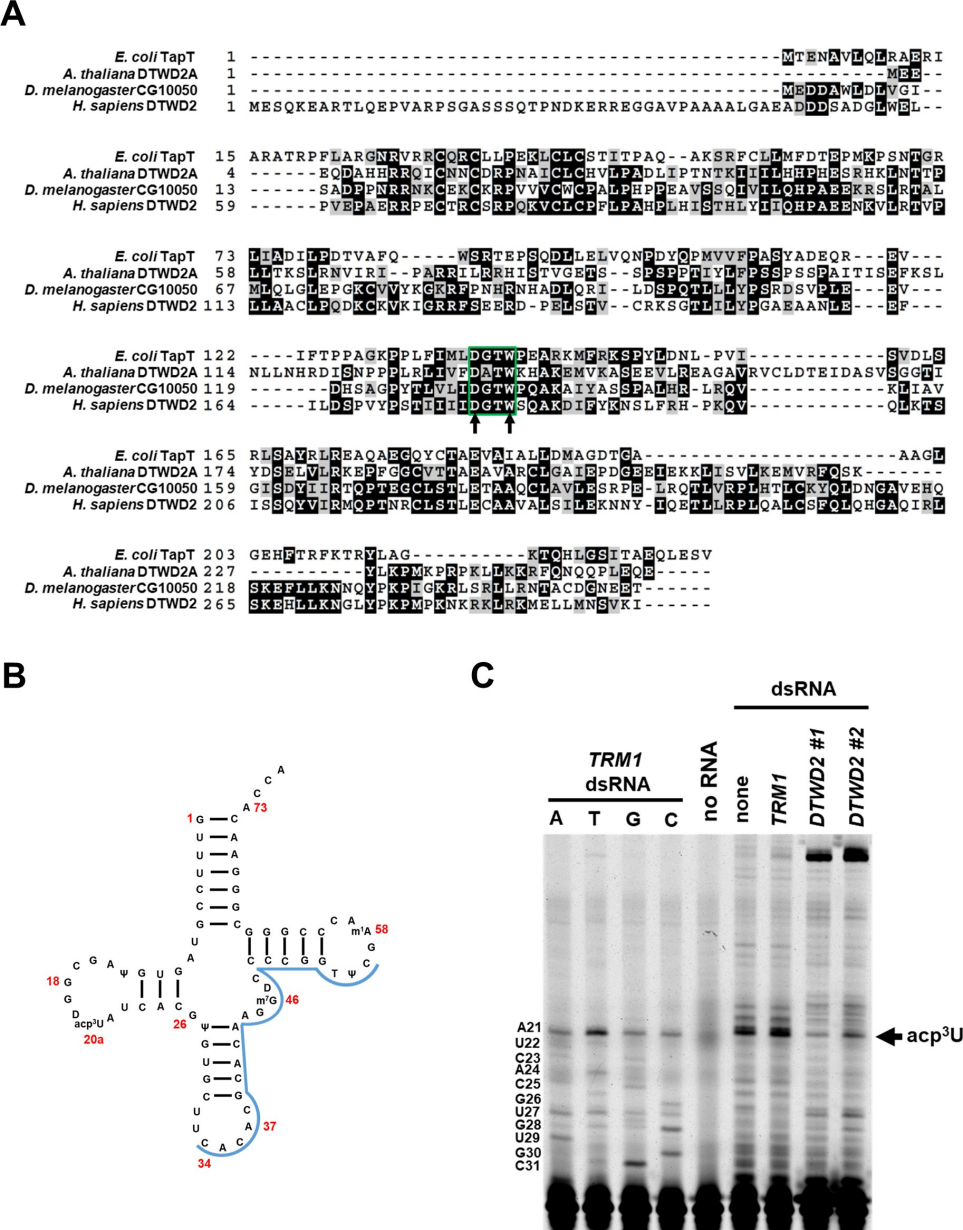
*CG10050* encodes the DTWD2A enzyme in *D*. *melanogaster*. **(A)** Sequence alignment of DTWD2 proteins. Green box represents the DTW domain, and arrows denote predicted active site residues. **(B)** Schematic of *D*. *melanogaster* tRNA^Val(CAC)^. Location of primer binding is shown in blue. Selected nucleotides are numbered in red. **(C)** Knockdown of *CG10050* by RNAi results in loss of a primer extension block consistent with acp^3^U_20b_ on tRNA. S2R+ cells were treated three times over 8 days with dsRNA to indicated genes. After harvest of cells, RNA was extracted and primer extension to tRNA^Val(CAC)^ was performed.

DTWD proteins were recently identified as the enzymes responsible for acp^3^U modifications on eukaryotic tRNA [76]. DTWD2 was shown to be responsible for the acp^3^U_20a_ tRNA modification in human cells, and homologs of these genes in other organisms were also identified by sequence [76]. We used our fluorescent primer extension approach to determine if DTWD2 genes are responsible for the acp^3^U_20a_ modification found on tRNA from *D*. *melanogaster* and for the acp^3^U_20b_ modification found on *A*. *thaliana* [1]. To this end, we silenced *CG10050*, which is the predicted DTWD2 homolog in *D*. *melanogaster* [76] (Fig 5A), and determined acp^3^U_20a_ modification levels on tRNA^Val(CAC)^ from these cells by primer extension (Fig 5B). A robust primer extension block on tRNA^Val(CAC)^ of a size consistent with that expected for acp^3^U_20a_ was observed on tRNA from untreated cells or cells treated with *TRM1* dsRNA (Fig 5C). In contrast, treatment of S2R+ cells with either of two different dsRNA constructs directed to *D*. *melanogaster DTWD2* resulted in a significant loss of the primer extension block (Fig 5C). No obvious difference in cell growth or morphology was observed between S2R+ cells treated with either *DTWD2* dsRNA construct and untreated cells. These results strongly suggest that *D*. *melanogaster DTWD2* forms acp^3^U_20a_ in *D*. *melanogaster* cells.

**Fig 6:**
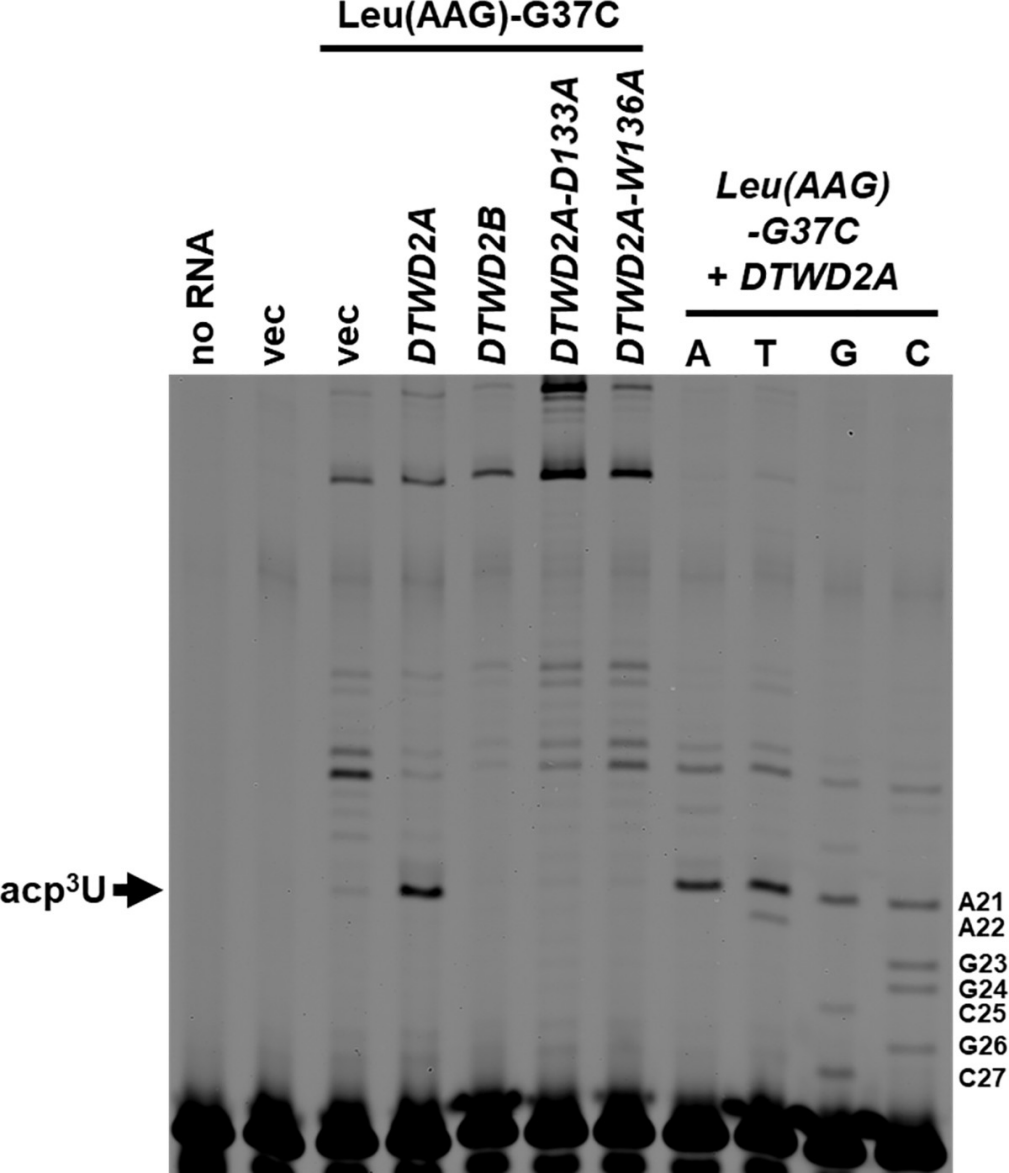
*A*. *thaliana* DTW2A expression results in a primer extension block consistent with acp^3^U_20b_ on *A*. *thaliana* tRNA. Bulk RNA was extracted from a *trm1Δ met22Δ* yeast strain expressing the indicated plasmids and then analyzed by primer extension to *A*. *thaliana* tRNA^Leu(AAG)^-G37C.

We next sought to identify the *A*. *thaliana* enzyme responsible for acp^3^U_20b_ formation on tRNA. In *A*. *thaliana*, DTWD2A (AT2G41750) and DTWD2B (AT5G54880) are the putative homologs of human DTWD2 [76] (Fig 5A). We therefore expressed each gene in *trm1Δ met22Δ* yeast cells with *A*. *thaliana* tRNA^Leu(AAG)^-G37C, which is expected to receive the acp^3^U_20b_ modification, because tRNA^Leu(AAG)^ from *L*. *luteus* contains acp^3^U_20b_ [95]. We used yeast cells containing the *trm1Δ* mutation to ensure that yeast Trm1 would not form the base-pair blocking m^2,2^G_26_ modification, and cells containing the *met22Δ* mutation to inhibit the rapid tRNA decay pathway [37]. We extracted RNA from the yeast cells and detected the formation of acp^3^U_20b_ by fluorescent primer extension. We found that expression of DTWD2A resulted in a primer extension block on tRNA^Leu(AAG)^-G37C when both the gene and the tRNA were expressed in yeast, consistent with the size expected for the acp^3^U_20b_ modification (Fig 6). This primer extension block was absent in yeast cells expressing only the tRNA. The block was also not present within the limits of our detection in cells expressing DTWD2B and the tRNA (Fig 6, Table 7).

**Table 7:** Relative mRNA levels of *A*. *thaliana DTWD2* genes expressed in yeast.

*A*. *thaliana* gene	target mRNA	relative levels^a^
*DTW2A*	*DTW2A*	25.38 ± 2.05
*DTWD2A-D133A*	*DTWD2A*	12.12 ± 5.19
*DTWD2A-W136A*	*DTWD2A*	17.23 ± 7.35
*DTW2B*	*DTW2B*	39.23 ± 7.35

^a^Relative to *S*. *cerevisiae* tubulin (*TUB1*), after both genes were normalized to *S*. *cerevisiae* actin (*ACT1*). Values are from three independent growths.

To verify that the primer extension block at position 21 on tRNA^Leu(AAG)^-G37C was due to the acp^3^U_20b_ modification, we performed LC-MS/MS on nucleosides from purified tRNA. tRNA nucleosides from *trm1Δ mett22Δ* cells expressing tRNA^Leu(AAG)^-G37C with or without *DTWD2A* were analyzed by reverse phased LC coupled with a triple quadrupole mass spectrometer. Selected reaction monitoring (SRM) was used to monitor the mass transitions for acp^3^U and U. We readily detected acp^3^U on tRNA^Leu(AAG)^-G37C purified from cells expressing DTWD2A, whereas levels of the modification on tRNA from cells expressing the vector control were near background levels (S5 Fig). To further verify that the observed LC peak was indeed acp^3^U, the nucleoside digest from the DTWD2A expressing cells was compared to a total *E*. *coli* tRNA nucleoside digest. *E*. *coli* contains several tRNAs with the acp^3^U modification [1]. The chromatogram and MS/MS spectra of acp^3^U from purified tRNA^Leu(AAG)^-G37C match the retention time and fragment ratio of acp^3^U from total *E*. *coli* tRNA (S6 and S7 Figs). Thus, we conclude that the primer extension block formed on tRNA^Leu(AAG)^-G37C in the presence of DTWD2A is due to acp^3^U.

These data suggest that DTWD2A forms acp^3^U_20b_ on plant tRNA, and are the first demonstration of an enzyme capable of forming an acp^3^U modification at position 20b on a tRNA. This result is somewhat surprising, because *A*. *thaliana* DTWD2A is more closely related to human DTWD2 than is *A*. *thaliana* DTWD2B [76]. Whether DTWD2A forms the modification in plant cells, and whether it can also form acp^3^U_20a_ is not clear. Additionally, we note that it is possible that DTWD2B is also capable of forming acp^3^U_20b_, but that the protein is not expressed well in yeast, or that it is incorrectly targeted in yeast cells.

DTWD2 belongs to the TDD superfamily of proteins, and is localized to the nucleus and the cytoplasm [78, 106, 107]. This protein superfamily contains *E*. *coli* TuaA/TapT, as well as Tsr3, which is required to add the acp group to the 1-methyl-3-(3-amino-3-carboxypropyl)-pseudouridine modification (m^1^acp^3^Ψ) found at residue U_1191_ on the yeast 18s rRNA and residue U_1248_ on human 18s rRNA. [78, 107]. TDD superfamily proteins contain a DTW domain with an E/DXS/TW motif [78, 106] (Fig 5A). In previously studied TDD proteins, the Asp/Glu and Trp residues of the motif are found in the active site and are required for catalysis. Indeed, Asp137 of this motif in *E*. *coli* TuaA/TapT is required for activity, and is likely the catalytic base, while Trp140 is required for activity and S-adenosyl methionine (SAM) cofactor binding [78, 107]. To determine if the corresponding Asp and Trp residues of the motif are required for the activity of a eukaryotic DTWD2 protein (Fig 5A), we generated D133A and W136A variants of *A*. *thaliana* DTW2a and tested their modification activity. We found that coexpression of either the DTWD2A-D133A or DTWD2A-W136A variants in yeast with tRNA^Leu(AAG)^-G37C did not result in the formation of a primer extension block consistent with acp^3^U_20b_ (Fig 6, Table 7), whereas expression of wild type DTWD2A resulted in formation of the primer extension block. These results further suggest that DTWD2A is responsible for the acp^3^U_20b_ modification found on *A*. *thaliana* tRNA. Because an in vitro assay for eukaryotic DTWD2 has not been reported, these results are the first experimental evidence that the Asp and Trp residues of the E/DXS/TW motif of a eukaryotic acp^3^U tRNA modification enzyme are required for activity. Whether *A*. *thaliana* DTWD2B and/or DTWD2A are required for the acp^3^U_20a_ modification of cytosolic tRNA, and whether the *A*. *thaliana* TapT-like enzyme is required for acp_3_U_47_ modification on plastid tRNA remains to be determined [76].

## Supporting information

S1 Fig Sequence alignment of TRM1 proteins. Green arrow denotes location of predicted active site aspartate residue.
(PDF)

S2 Fig *A*. *thaliana* TRM1a and TRM1b form m^2,2^G on *A*. *thaliana* tRNA^Leu(AAG)^. Overlay of extracted ion chromatograms showing the abundance of m^2,2^G (*m/z* 312.131 ± 5 ppm) on *A*. *thaliana* tRNA^Leu(AAG)^-G37C expressed in *trm1Δ* mutant yeast expressing TRM1a (green), TRM1b (red), or a vector control (blue).
(PDF)

S3 Fig Sequence alignment of TRM1c homologs in various plants species. (PDF)

S4 Fig Knockdown of *CG6388* by RNAi results in loss of a primer extension block consistent with m^2,2^G_26_ and the appearance of a block consistent with acp^3^U_20_. S2R+ cells were treated twice over 6 days with dsRNA to indicated gene as described in Materials and methods. After harvest of cells, RNA was extracted and primer extension to tRNA^Tyr^ was performed. Sequencing was performed on cells treated with dsRNA to *CG6388* to determine the location of the new primer extension block which appeared upon loss of the m^2,2^G_26_ block.
(PDF)

S5 Fig *A*. *thaliana* DTWD2A is necessary for acp^3^U modification on *A*. *thaliana* tRNA^Leu(AAG)^. Chromatogram overlays of acp^3^U (top) and uridine (bottom) from *A*. *thaliana* tRNA^Leu(AAG)^-G37C expressed in yeast cells expressing DTWD2A (red) or a vector control (blue). Equal amounts of tRNA from indicated samples were analyzed by nucleoside LC-MS/MS. The abundance of acp^3^U (top; mass transition 346 → 214, 346 → 197, 346 → 168) and uridine (bottom; mass transition 245 → 113) are shown.
(PDF)

S6 Fig Comparison of acp^3^U chromatograms from *E*. *coli* tRNA nucleosides and *A*. *thaliana* tRNA^Leu(AAG)^ nucleosides. Chromatograms of acp^3^U (mass transition 346 → 214, 346 → 197, 346 → 168) from *E*. *coli* tRNA nucleosides (black) and *A*. *thaliana* tRNA^Leu(AAG)^-G37C nucleosides from cells expressing DTWD2A (red) are shown.
(PDF)

S7 Fig Comparison of MS/MS of acp^3^U in *E*. *coli* tRNA nucleosides and *A*. *thaliana* tRNA^Leu(AAG)^ nucleosides. MS/MS of acp^3^U (mass transition 346 → 214, 346 → 197, 346 → 168) from *E*. *coli* tRNA nucleosides (top) and *A*. *thaliana* tRNA^Leu(AAG)^-G37C nucleosides from cells expressing DTWD2A (bottom) are shown.
(PDF)

S1 Raw images (PDF)
