# Supplementary material for: Identification of the enzymes responsible for m2,2G and acp3U formation on cytosolic tRNA from insects and plants
Source: PLoS One. 2020 Nov 30;15(11):e0242737. doi: 10.1371/journal.pone.0242737 (PMC7704012; doi:10.1371/journal.pone.0242737)
Supplement: S1 Fig — Green arrow denotes location of predicted active site aspartate residue. (PDF) [file pone.0242737.s001.pdf]

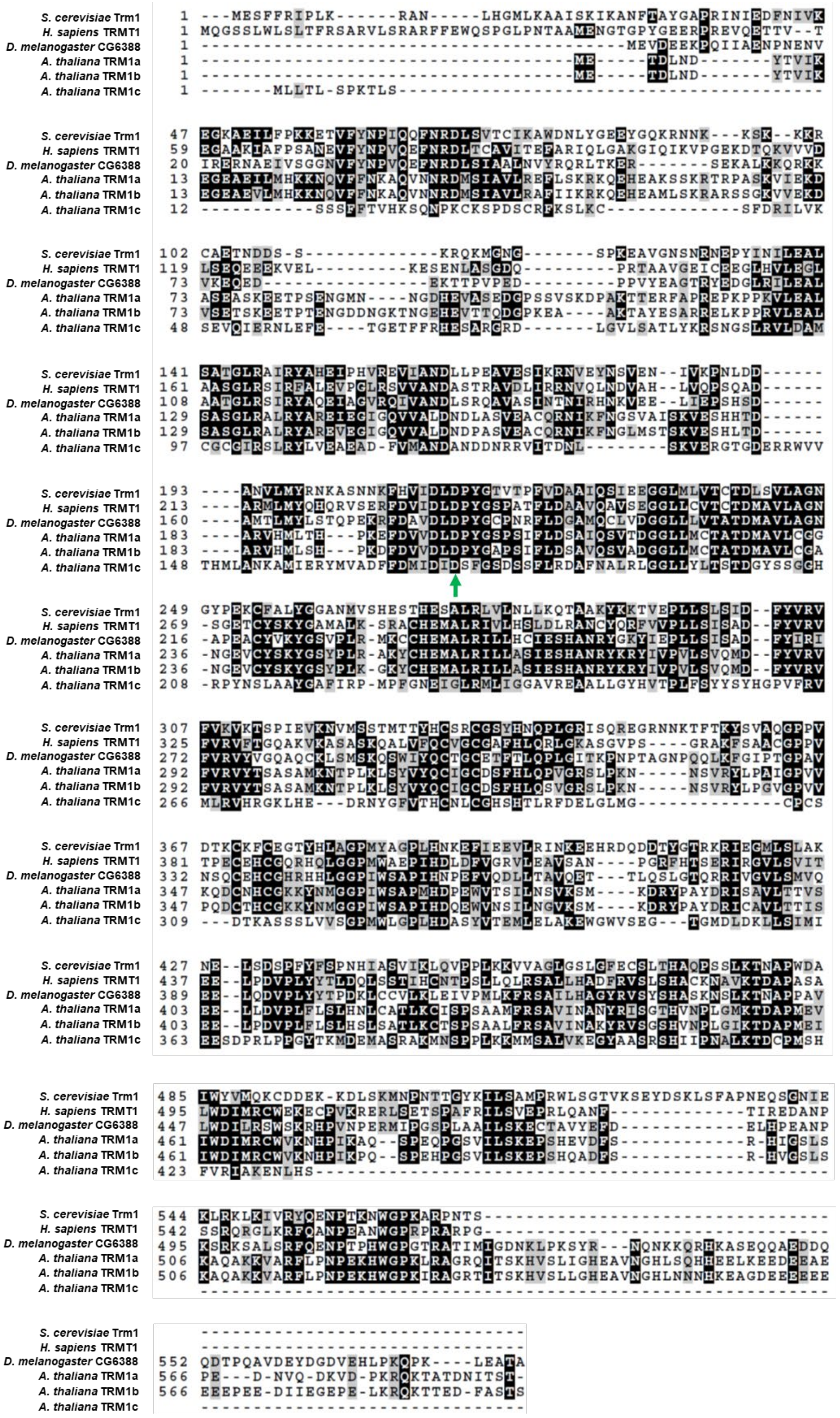
S. cerevisiae Trm1 1 ---MESFFRIPLK------RAN-----LHGMLKAAISKIKANFTAYGAPRINIEDFNIVK
H. sapiens TRMT1 1 MQGSSLWLSLTFRSARVLSRARFFEWQSPGLPNTAAMENGTGPYGEERPREVQETTV--T
D. melanogaster CG6388 1 --MEVDEEKPQIIAENPNENV
A. thaliana TRM1a 1 ------------------------------------ME-----TDLND-------YTVIK
A. thaliana TRM1b 1 ------------------------------------ME-----TDLND-------YTVIK
A. thaliana TRM1c 1 -------MLLTL-SPKTLS--
S. cerevisiae Trm1 47 EGKAEILFPKKETVFYNPIQQFNRDLSVTCIKAWDNLYGEEYGQKRNNK---KSK--KKR
H. sapiens TRMT1 59 EGAAKIAFPSANEVFYNPVQEFNRDLTCAVITEFARIQLGAKGIQIKVPGEKDTQKVVVD
D. melanogaster CG6388 20 IRERNAEIVSGGNVFYNPVQEFNRDLSIAALNVYRQRLTKER-------SEKALKKQRKK
A. thaliana TRM1a 13 EGEAEILMHKKNQVFFNKAQVNNRDMSIAVLREFLSKRKQEHEAKSSKRTRPASKVIEKD
A. thaliana TRM1b 13 EGEAEVLMHKKNQVFFNKAQVNNRDMSIAVLRAFIIKRKQEHEAMLSKRARSSGKVVEKD
A. thaliana TRM1c 12 -----------SSSFFTVHKSQNPKCKSPDSCRFKSLKC------------SFDRILVK
S. cerevisiae Trm1 102 CAETNDDS-S-------------KRQKMGNG-------SPKEAVGNSNRNEPYINILEAL
H. sapiens TRMT1 119 LSEQEEEKVEL---------KESENLASGDQ---------PRTAAVGEICEEGLHVLEGL
D. melanogaster CG6388 73 VKEQED---------------EKTTPVPED---------PPVYEAGTRYEDGLRILEAL
A. thaliana TRM1a 73 ASEASKEETPSENGMN----NGDHEVASEDGPSSVSKDPAKTTERFAPREPKPPKVLEAL
A. thaliana TRM1b 73 VSETSKEETPTENGDDNGKTNGEHEVTTQDGPKEA----AKTAYESARRELKPPRVLEAL
A. thaliana TRM1c 48 SEVQIERNLEFE----TGETFFRHESARGRD-------LGVLSATLYKRSNGSLRVLDAM
S. cerevisiae Trm1 141 SATGLRAIRYAHEIPHVREVIANDLLPEAVESIKRNVEYNSVEN--IVKPNLDD------
H. sapiens TRMT1 161 AASGLRSIRFALEVPGLRSVVANDASTRAVDLIRRNVQLNDVAH--LVQPSQAD------
D. melanogaster CG6388 108 AATGLRSIRYAQEIAGVRQIVANDLSRQAVASINTNIRHNKVEE--LIEPSHSD------
A. thaliana TRM1a 129 SASGLRALRYAREIEGIGQVVALDNDLASVEACQRNIKFNGSVAISKVESHHTD------
A. thaliana TRM1b 129 SASGLRALRYAREVEGIGQVVALDNDPASVEACQRNIKFNGLMSTSKVESHLTD------
A. thaliana TRM1c 97 CGCGIRSLRYLVEAEAD-FVMANDANDDNRRVITDNL--------SKVERGTGDERRWVV
S. cerevisiae Trm1 193 ----ANVLMYRNKASNNKFHVIDLDPYGTVTPFVDAAIQSIEEGGLMLVTCTDLSVLAGN
H. sapiens TRMT1 213 ----ARMLMYQHQRVSERFDVIDLDPYGSPATFLDAAVQAVSEGGLLCVTCTDMAVLAGN
D. melanogaster CG6388 160 ----AMTLMYLSTQPEKRFDAVDLDPYGCPNRFLDGAMQCLVDGGLLLVTATDMAVLAGN
A. thaliana TRM1a 183 ----ARVHMLTH---PKEFDVVDLDPYGSPSIFLDSAIQSVTDGGLLMCTATDMAVLCGG
A. thaliana TRM1b 183 ----ARVHMLSH---PKDFDVVDLDPYGAPSIFLDSAVQSVADGGLLMCTATDMAVLCGA
A. thaliana TRM1c 148 THMLANKAMIERYMVADFFDMIDIDSFGSDSSFLRDAFNALRLGGLLYLTSTDGYSSGGH
S. cerevisiae Trm1 249 GYPEKCFALYGGANMVSHESTHESALRLVLNLLKQTAAKYKKTVEPLLSLSID--FYVRV
H. sapiens TRMT1 269 -SGETCYSKYGAMALK-SRACHEMALRIVLHSLDLRANCYQRFVVPLLSISAD--FYVRV
D. melanogaster CG6388 216 -APEACYVKYGSVPLR-MKCCHEMALRILLHCIESHANRYGKYIEPLLSISAD--FYIRI
A. thaliana TRM1a 236 -NGEVCYSKYGSYPLK-AKYCHEMALRILLASIESHANRYKRYIVPVLSVQMD--FYVRV
A. thaliana TRM1b 236 -NGEVCYSKYGSYPLK-GKYCHEMALRILLASIESHANRYKRYIVPVLSVQMD--FYVRV
A. thaliana TRM1c 208 -RPYNSLAAYGAFIRP-MPFGNEIGLRMLIGGAVREAALLGYHVTPLFSYYSYHGPVFRV
S. cerevisiae Trm1 307 FVKVKTSPIEVKNVMSSTMTTYHCSRCGSYHNQPLGRISQREGRNNKTFTKYSVAQGPPV
H. sapiens TRMT1 325 FVRVFTGQAKVKASASKQALVFQCVGCGAFHLQRLGKASGVPS----GRAKFSAACGPPV
D. melanogaster CG6388 272 FVRVYVGQAQCKLSMSKQSWIYQCTGCETFTLQPLGITKPNPTAGNPQQLKFGIPTGPAV
A. thaliana TRM1a 292 FVRVYTSASAMKNTPLKLSYVYQCIGCDSFHLQPVGRSLPKN-----NSVRYLPAIGPVV
A. thaliana TRM1b 292 FVRVYTSASAMKNTPLKLSYVYQCIGCDSFHLQSVGRSLPKN-----NSVRYLPGVGPVV
A. thaliana TRM1c 266 MLRVHRGKLHE---DRNYGFVTHCNLCGHSHTLRFDELGLMG-------------CPCS
S. cerevisiae Trm1 367 DTKCKFCEGTYHLAGPMYAGPLHNKEFIEEVLRINKEEHRDQDDTYGTRKRIEGMLSLAK
H. sapiens TRMT1 381 TPECEHCGQRHQLGGPMWAEPIHDLDFVGRVLEAVSAN----PGRFHTSERIRGVLSVIT
D. melanogaster CG6388 332 NSQCEHCGHRHHLGGPIWSAPIHNPEFVQDLLTAVQET---TLQSLGTQRRIVGVLSMVQ
A. thaliana TRM1a 347 KQDCNHCGKKYNMGGPIWSAPMHDPEWVTSILNSVKSM----KDRYPAYDRISAVLTTVS
A. thaliana TRM1b 347 PQDCTHCGKKYNMGGPIWSAPIHDQEWVNSILNGVKSM----KDRYPAYDRICAVLTTIS
A. thaliana TRM1c 309 ---DTKASSSLVVSGPMWLGPLHDASYVTEMLELAKEWGWVSEG---TGMDLDKLLSIMI
S. cerevisiae Trm1 427 NE--LSDSPFYFSPNHIASVIKLQVPPLKKVVAGLGSLGFECSLTHAQPSSLKTNAPWDA
H. sapiens TRMT1 437 EE--LPDVPLYYTLDQLSSTIHCNTPSLLQLRSALLHADFRVSLSHACKNAVKTDAPASA
D. melanogaster CG6388 389 EE--LQDVPLYYTPDKLCCVLKLEIVPMLKFRSAILHAGYRVSYSHASKNSLKTNAPPAV
A. thaliana TRM1a 403 EE--LLDVPLFLSLHNLCATLKCISPSAAMFRSAVINANYRISGTHVNPLGMKTDAPMEV
A. thaliana TRM1b 403 EE--LPDVPLFLSLHSLSATLKCTSPSAALFRSAVINAKYRVSGSHVNPLGIKTDAPMEI
A. thaliana TRM1c 363 EESDPRLPPGYTKMDEMASRAKMNSPPLKKMMSALVKEGYAASRSHIIPNALKTDCPMSH
S. cerevisiae Trm1 485 IWYVMQKCDDEK-KDLSKMNPNTTGYKILSAMPRWLSGTVKSEYDSKLSFAPNEQSGNIE
H. sapiens TRMT1 495 LWDIMRCWEKECPVKRERLSETSPAFRILSVEPRLQANF-------------TIREDANP
D. melanogaster CG6388 447 LWDILRSWSKRHPVNPERMIPGSPLAAILSKECTAVYEFD------------ELHPEANP
A. thaliana TRM1a 461 IWDIMRCWVKNHPIKAQ--SPEQPGSVILSKEPSHEVDFS------------R-HIGSLS
A. thaliana TRM1b 461 IWDIMRCWVKNHPIKPQ--SPEHPGSVILSKEPSHQADFS------------R-HVGSLS
A. thaliana TRM1c 423 FVRIAKENLHS---
S. cerevisiae Trm1 544 KLRKLKIVRYQENPTKNWGPKARPNTS---------------------------------
H. sapiens TRMT1 542 SSRQRGLKRFQANPEANWGPRPRARPG---------------------------------
D. melanogaster CG6388 495 KSRKSALSRFQENPTPHWGPGTRATIMIGDNKLPKSYR---NQNKKQRHKASEQQAEDDQ
A. thaliana TRM1a 506 KAQAKKVARFLPNPEKHWGPKLRAGRQITSKHVSLIGHEAVNGHLSQHHEELKEEDEEAE
A. thaliana TRM1b 506 KAQAKKVARFLPNPEKHWGPKIRAGRTITSKHVSLLGHEAVNGHLNNNHKEAGDEEEEEE
A. thaliana TRM1c --
S. cerevisiae Trm1 -------------------------------
H. sapiens TRMT1 -------------------------------
D. melanogaster CG6388 552 QDTPQAVDEYDGDVEHLPKQPK----LEATA
A. thaliana TRM1a 566 PE---D-NVQ-DKVD-PKRQKTATDNITST-
A. thaliana TRM1b 566 EEEPEE-DIIEGEPE-LKRQKTTED-FASTS
A. thaliana TRM1c -------------------------------
