# Supplementary figures and images for: Identification of the enzymes responsible for m2,2G and acp3U formation on cytosolic tRNA from insects and plants
Source: PLoS One. 2020 Nov 30;15(11):e0242737. doi: 10.1371/journal.pone.0242737 (PMC7704012; doi:10.1371/journal.pone.0242737)

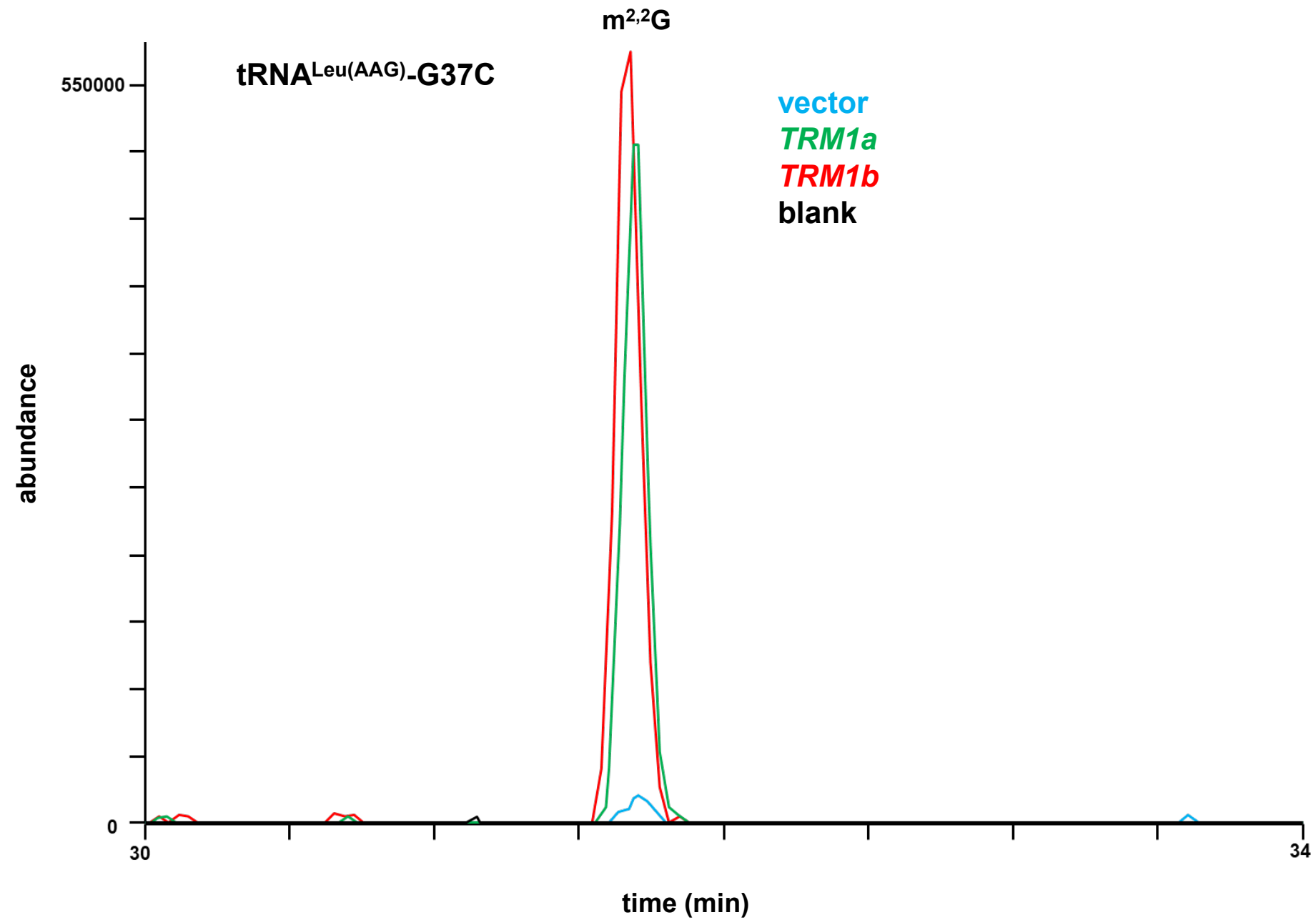

Supplement: S2 Fig — Overlay of extracted ion chromatograms showing the abundance of m2,2G (m/z 312.131 ± 5 ppm) on A. thaliana tRNALeu(AAG)-G37C expressed in trm1Δ mutant yeast expressing TRM1a (green), TRM1b (red), or a vector control (blue). (PDF) [file pone.0242737.s002.pdf]

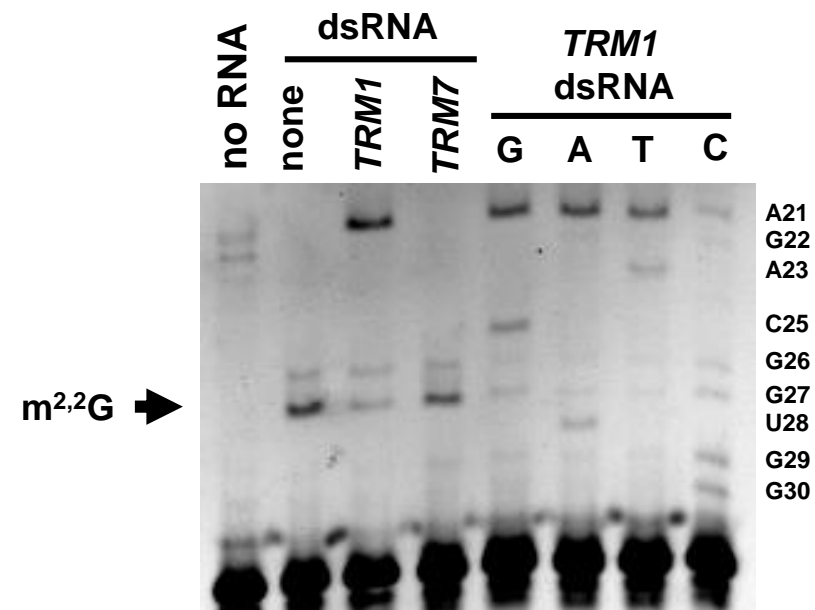

Supplement: S4 Fig — S2R+ cells were treated twice over 6 days with dsRNA to indicated gene as described in Materials and methods. After harvest of cells, RNA was extracted and primer extension to tRNATyr was performed. Sequencing was performed on cells treated with dsRNA to CG6388 to determine the location of the new primer extension block which appeared upon loss of the m2,2G26 block. (PDF) [file pone.0242737.s004.pdf]

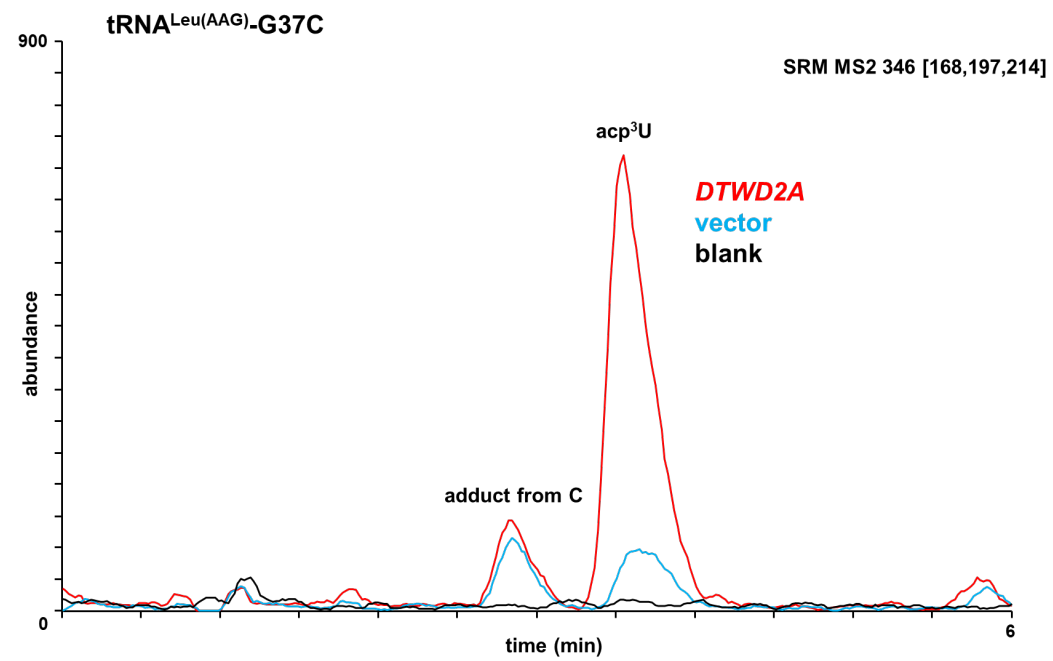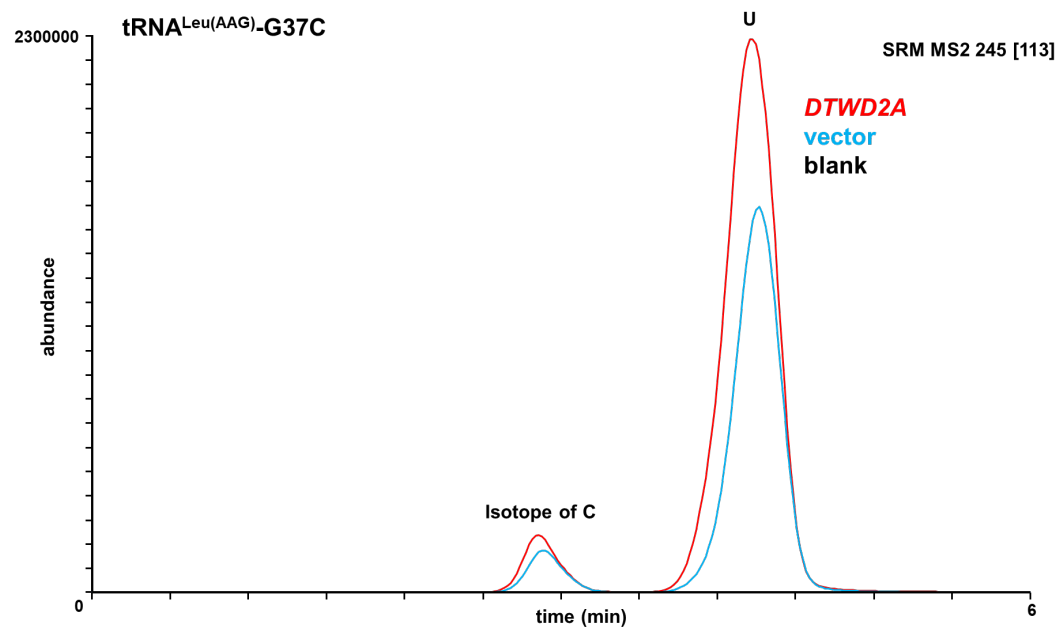

Supplement: S5 Fig — Chromatogram overlays of acp3U (top) and uridine (bottom) from A. thaliana tRNALeu(AAG)-G37C expressed in yeast cells expressing DTWD2A (red) or a vector control (blue). Equal amounts of tRNA from indicated samples were analyzed by nucleoside LC-MS/MS. The abundance of acp3U (top; mass transition 346 → 214, 346 → 197, 346 → 168) and uridine (bottom; mass transition 245 → 113) are shown. (PDF) [file pone.0242737.s005.pdf]

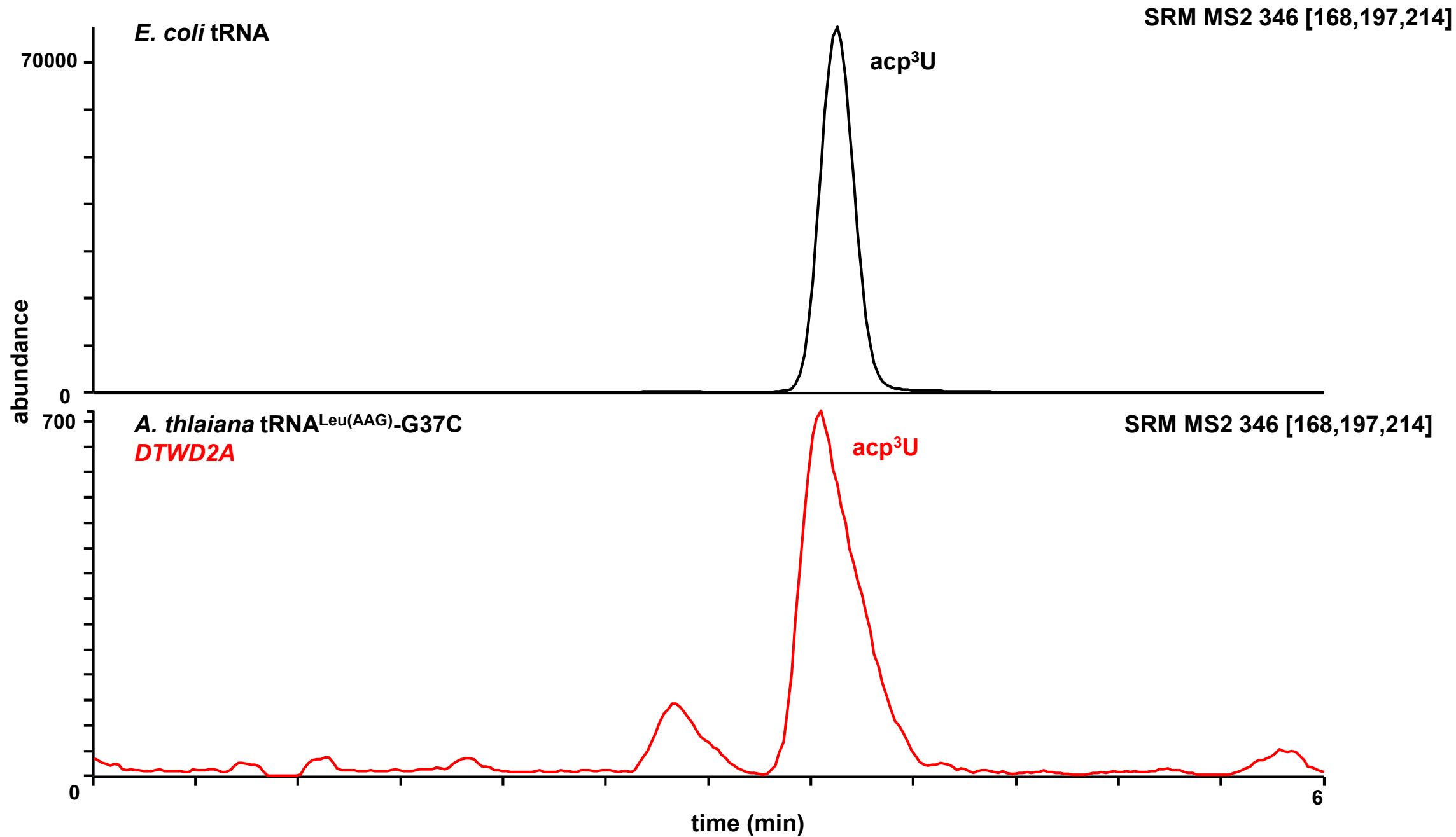

Supplement: S6 Fig — Chromatograms of acp3U (mass transition 346 → 214, 346 → 197, 346 → 168) from E. coli tRNA nucleosides (black) and A. thaliana tRNALeu(AAG)-G37C nucleosides from cells expressing DTWD2A (red) are shown. (PDF) [file pone.0242737.s006.pdf]

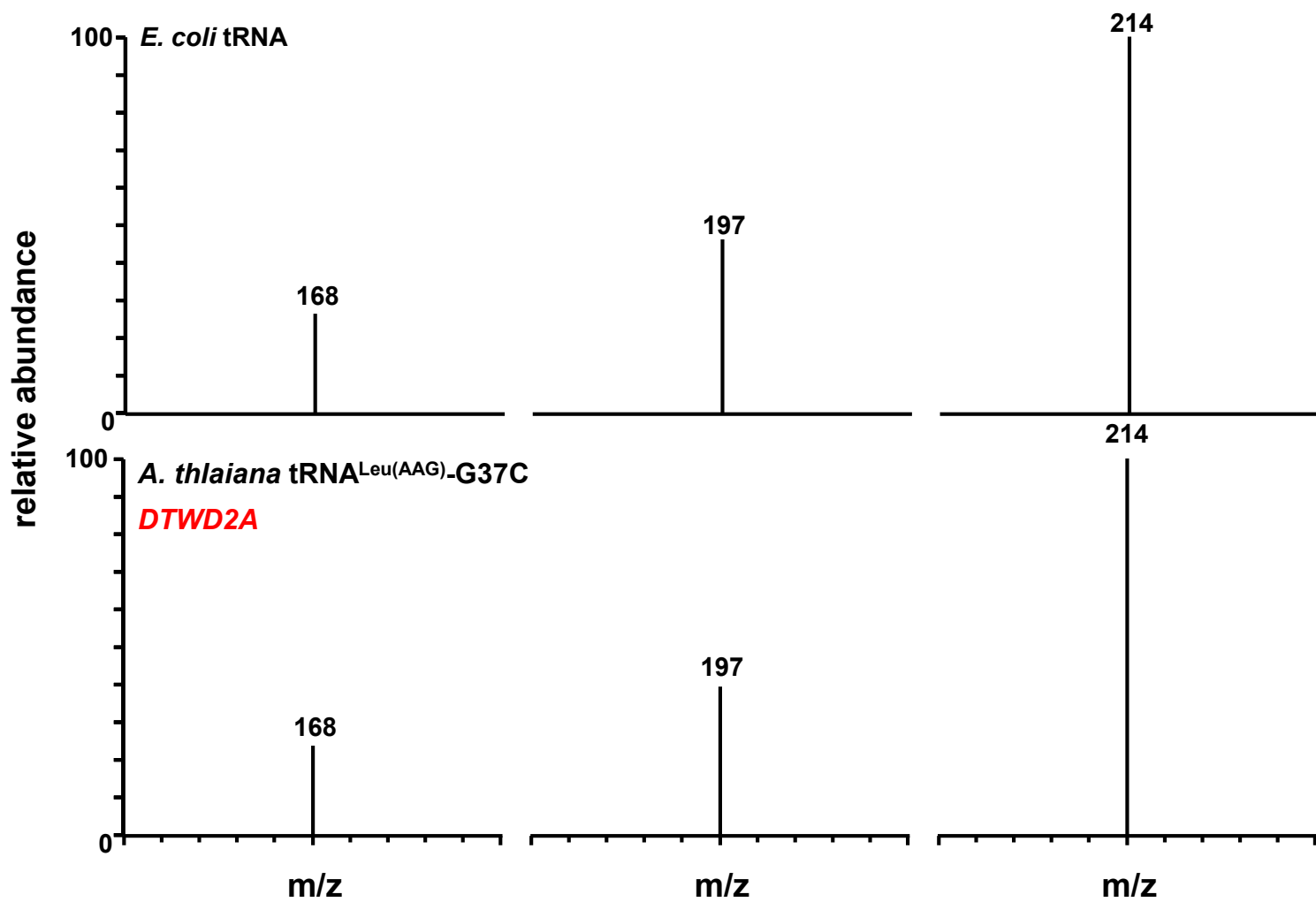

Supplement: S7 Fig — MS/MS of acp3U (mass transition 346 → 214, 346 → 197, 346 → 168) from E. coli tRNA nucleosides (top) and A. thaliana tRNALeu(AAG)-G37C nucleosides from cells expressing DTWD2A (bottom) are shown. (PDF) [file pone.0242737.s007.pdf]
