# Supplementary material for: Identification of the enzymes responsible for m2,2G and acp3U formation on cytosolic tRNA from insects and plants
Source: PLoS One. 2020 Nov 30;15(11):e0242737. doi: 10.1371/journal.pone.0242737 (PMC7704012; doi:10.1371/journal.pone.0242737)
Supplement: S3 Fig — (PDF) [file pone.0242737.s003.pdf]

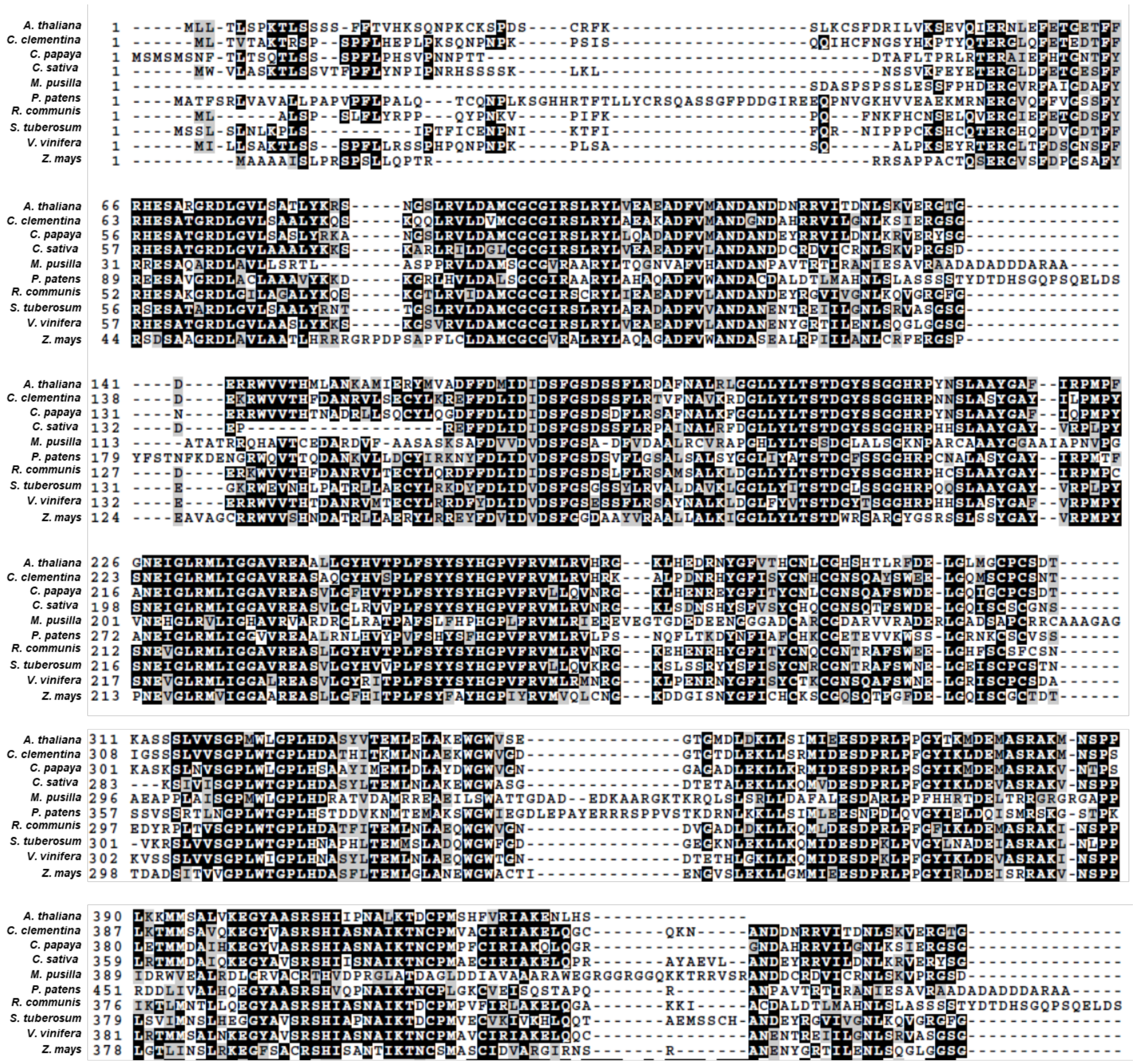

A. thaliana
C. clementina
C. papaya
C. sativa
M. pusilla
P. patens
R. communis
S. tuberosum
V. vinifera
Z. mays
