# Supplementary material for: Identification of the enzymes responsible for m2,2G and acp3U formation on cytosolic tRNA from insects and plants
Source: PLoS One. 2020 Nov 30;15(11):e0242737. doi: 10.1371/journal.pone.0242737 (PMC7704012; doi:10.1371/journal.pone.0242737)
Supplement: S1 Raw images — (PDF) [file pone.0242737.s008.pdf]

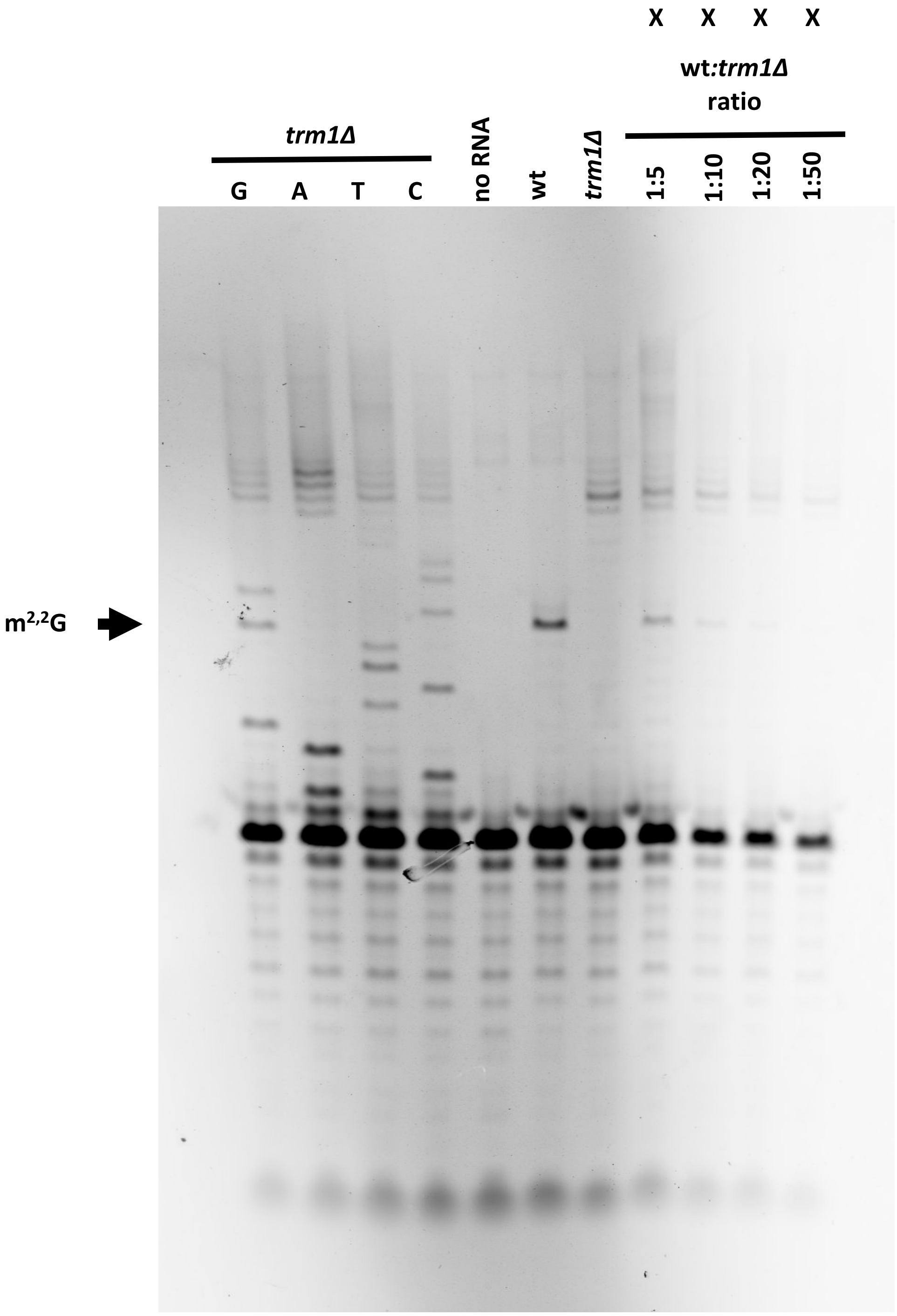

Fig. 2B(left). Detection of $m^{2,2}G_{26}$ by fluorescent primer extension in yeast cells.

Bulk RNA was extracted from culture of indicated individual strains or culture from mixtures of strains in indicated ratios, and then analyzed by primer extension to yeast $tRNA^{Tyr}$. 10 umol 5′ Tye665-labeled oligonucleotides (Integrated DNA Technologies) was annealed to 1.29 uL RNA, heated to 95°C and slow cooled to 37°C. The entire reaction was then incubated with 1 mM dNTPs and 1.89 U of Avian Myeloblastosis Virus (AMV). Sequencing reactions were annealed similarly and extended overnight with the addition of 0.1 mM ddNTP's. Reactions were incubated at 37°C overnight, and analyzed by 15% PAGE with 7M Urea. The gel was placed between overhead projector sheets and visualized using a Typhoon 9200 scanner with a 620 BP30 Cy5 emission filter at high sensitivity.

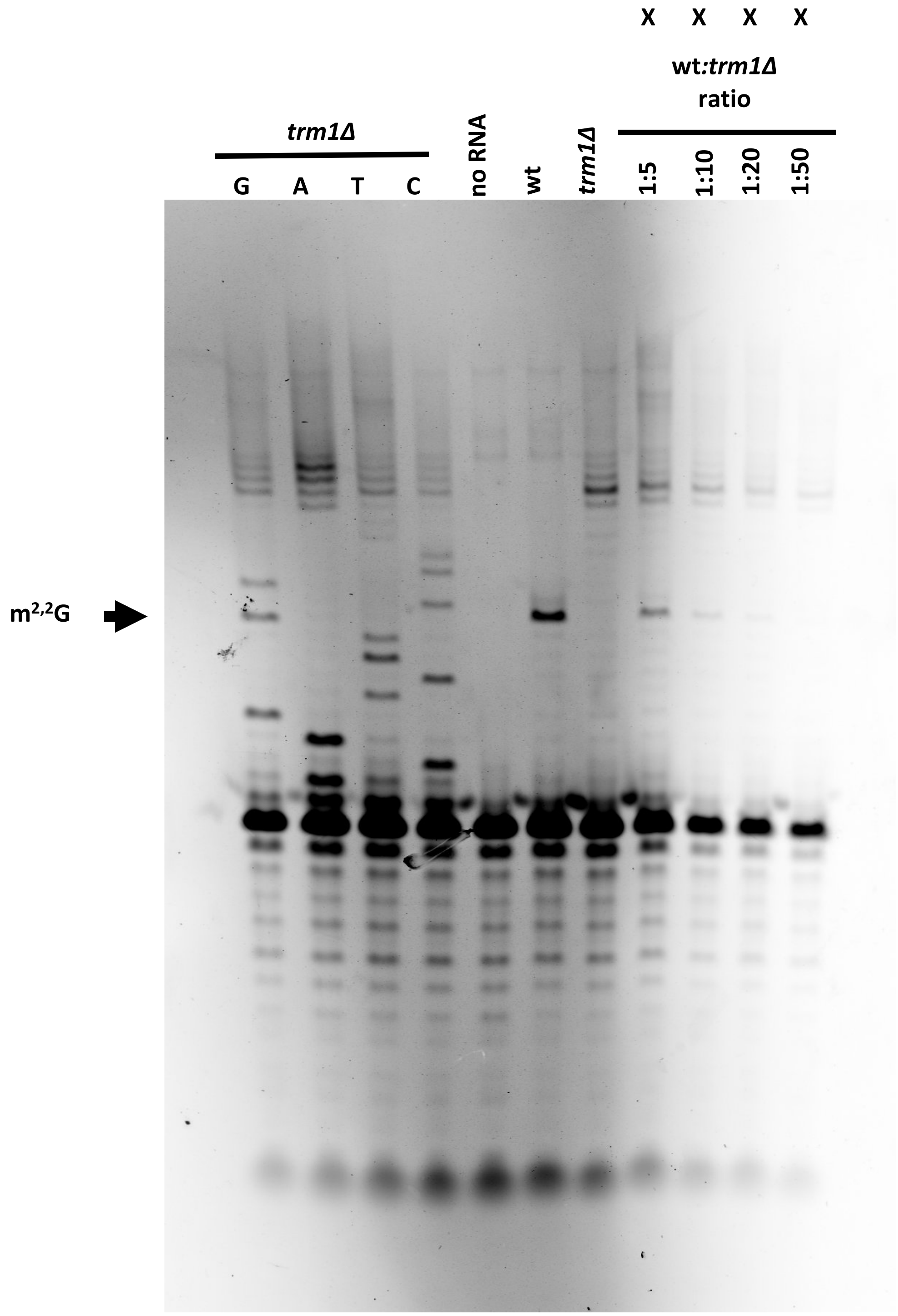

Fig. 2B(left). Detection of $m^{2,2}G_{26}$ by fluorescent primer extension in yeast cells.

Bulk RNA was extracted from culture of indicated individual strains or culture from mixtures of strains in indicated ratios, and then analyzed by primer extension to yeast $tRNA^{Tyr}$. 10 umol 5’ Tye665-labeled oligonucleotides (Integrated DNA Technologies) was annealed to 1.29 uL RNA, heated to 95°C and slow cooled to 37°C. The entire reaction was then incubated with 1 mM dNTPs and 1.89 U of Avian Myeloblastosis Virus (AMV). Sequencing reactions were annealed similarly and extended overnight with the addition of 0.1 mM ddNTP’s. Reactions were incubated at 37°C overnight, and analyzed by 15% PAGE with 7M Urea. The gel was placed between overhead projector sheets and visualized using a Typhoon 9200 scanner with a 620 BP30 Cy5 emission filter at high sensitivity. Brightness and contrast adjusted and applied equally to entire gel.

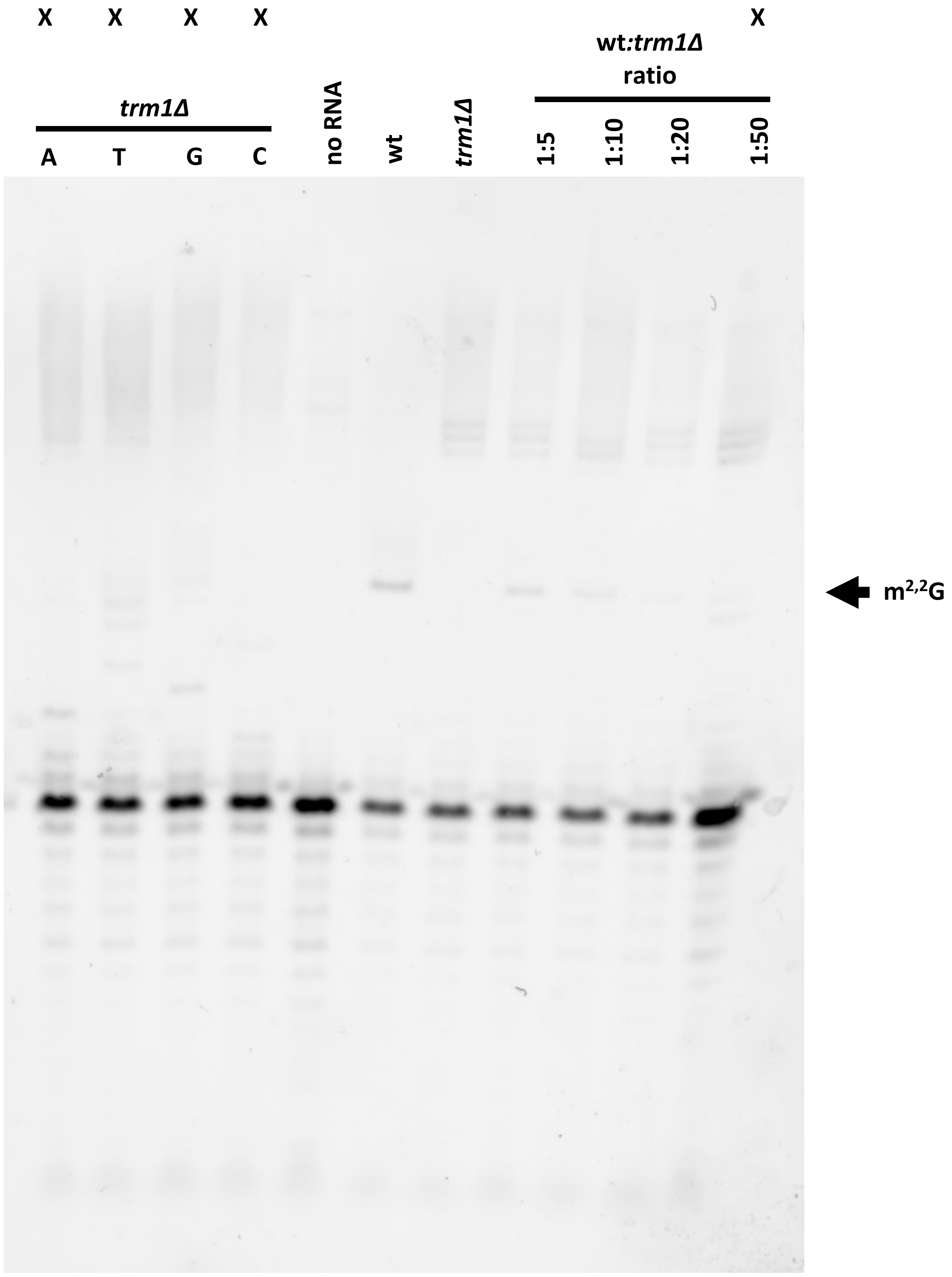

Fig. 2B(right). Detection of $m^{2,2}G_{26}$ by fluorescent primer extension in yeast cells.

Bulk RNA was extracted from culture of indicated individual strains or culture from mixtures of strains in indicated ratios, and then analyzed by primer extension to yeast $tRNA^{Tyr}$. 10 umol 5' Tye665-labeled oligonucleotides (Integrated DNA Technologies) was annealed to 3uL RNA, heated to 95°C and slow cooled to 37°C. The entire reaction was then incubated with 1 mM dNTPs and 1.89 U of Avian Myeloblastosis Virus (AMV). Sequencing reactions were annealed similarly and extended overnight with the addition of 0.1 mM ddNTP's. Reactions were incubated at 37°C overnight, and analyzed by 15% PAGE with 7M Urea. The gel was placed between overhead projector sheets and visualized using a Typhoon 9200 scanner with a 620 BP30 Cy5 emission filter at high sensitivity.

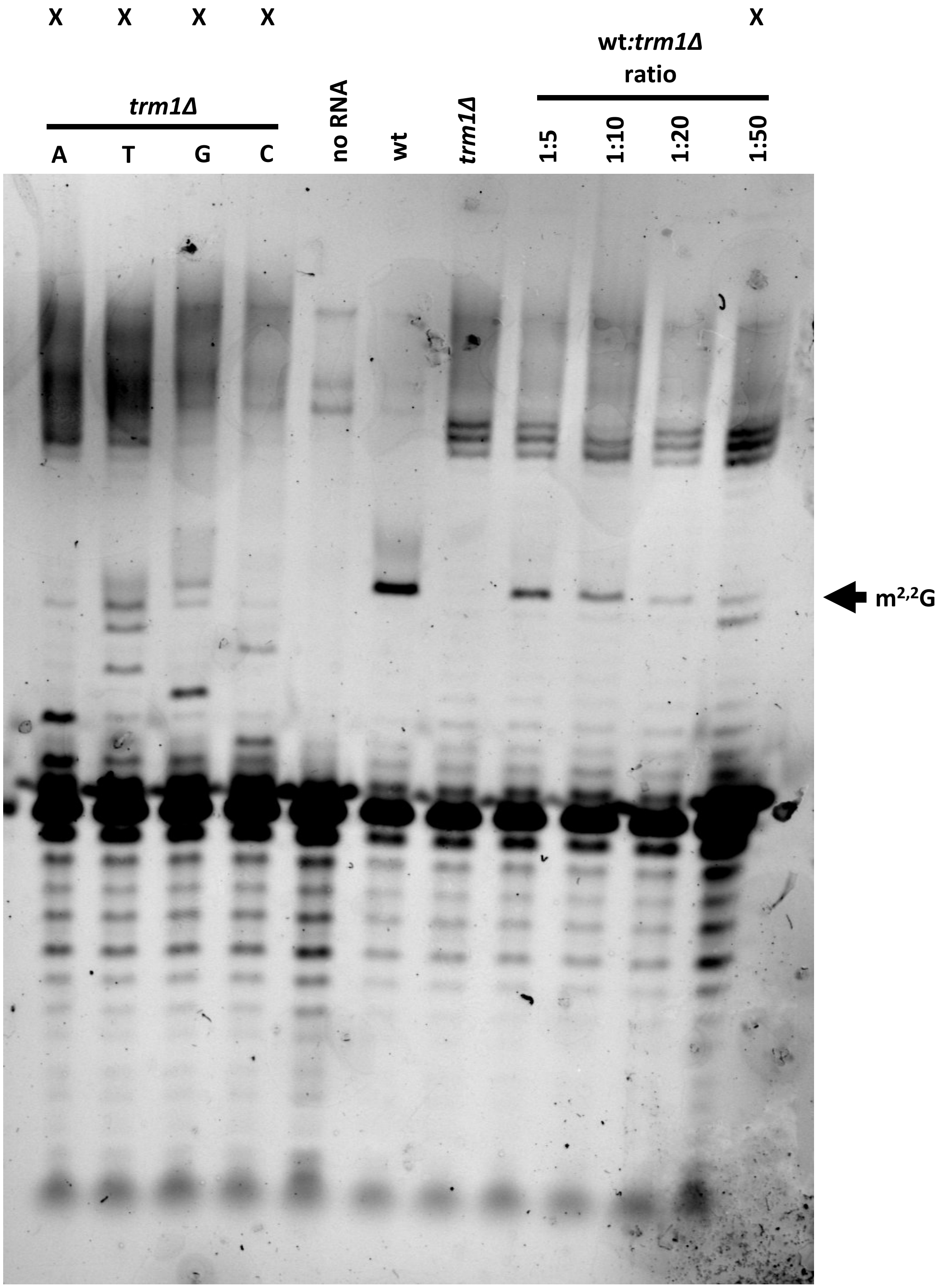

Fig. 2B(right). Detection of $m^{2,2}G_{26}$ by fluorescent primer extension in yeast cells.

Bulk RNA was extracted from culture of indicated individual strains or culture from mixtures of strains in indicated ratios, and then analyzed by primer extension to yeast $tRNA^{Tyr}$. 10 umol 5' Tye665-labeled oligonucleotides (Integrated DNA Technologies) was annealed to 3uL RNA, heated to 95°C and slow cooled to 37°C. The entire reaction was then incubated with 1 mM dNTPs and 1.89 U of Avian Myeloblastosis Virus (AMV). Sequencing reactions were annealed similarly and extended overnight with the addition of 0.1 mM ddNTP's. Reactions were incubated at 37°C overnight, and analyzed by 15% PAGE with 7M Urea. The gel was placed between overhead projector sheets and visualized using a Typhoon 9200 scanner with a 620 BP30 Cy5 emission filter at high sensitivity. Brightness and contrast adjusted and applied equally to entire gel.

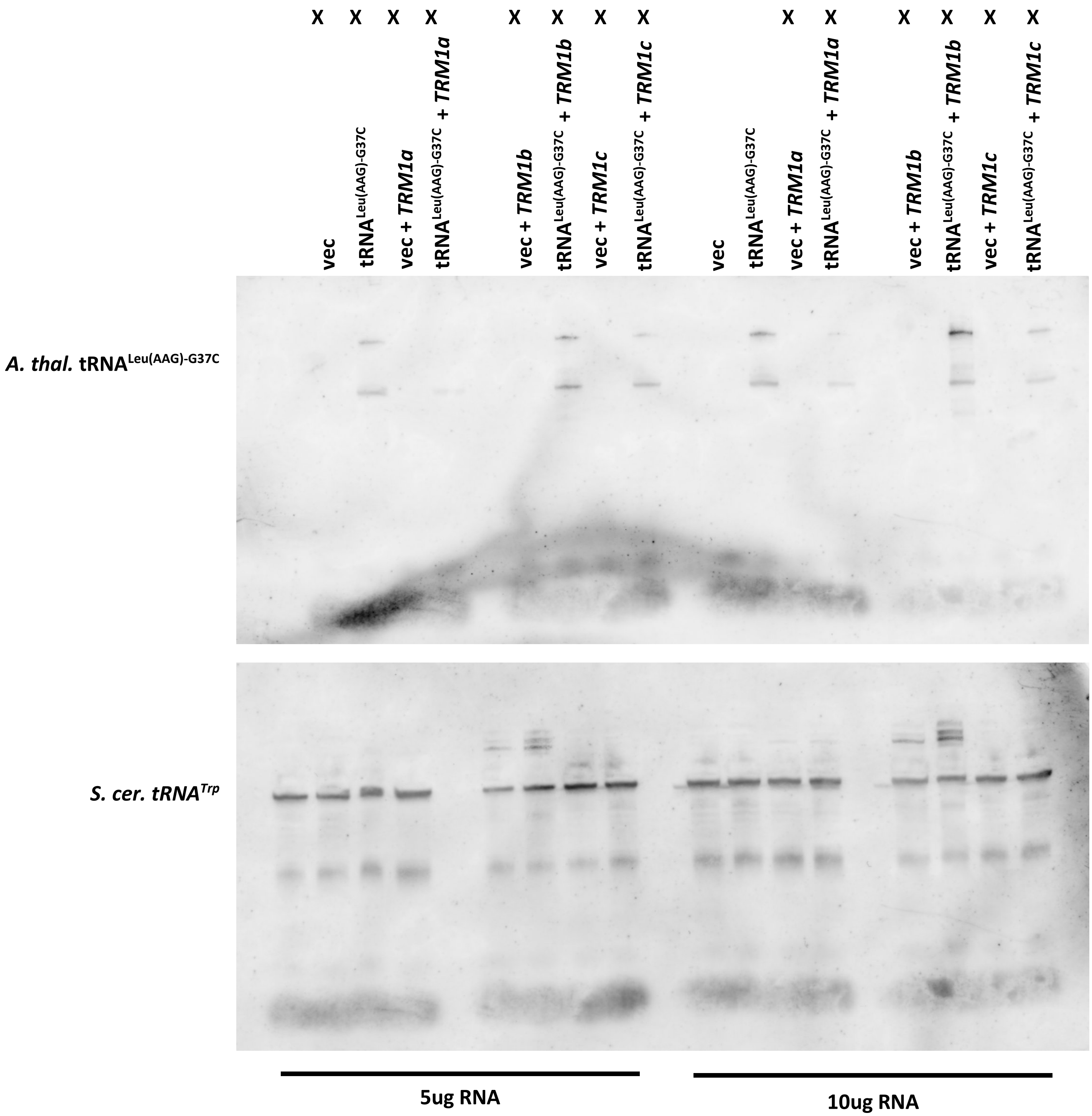

Fig. 3B. *A. thaliana* tRNA$^{Leu(AAG)}$-G37C is expressed in yeast cells.

RNA was extracted from indicated strains and analyzed by Northern blot. 5ug and 10ug of bulk RNA were analyzed by 10% PAGE and transferred to a nitrocellulose membrane. 5' Tye665-labeled oligonucleotide probes specific to the indicated tRNA were used for detection. The gel was visualized using a Typhoon 9200 scanner with a 620 BP30 Cy5 emission filter at high sensitivity.

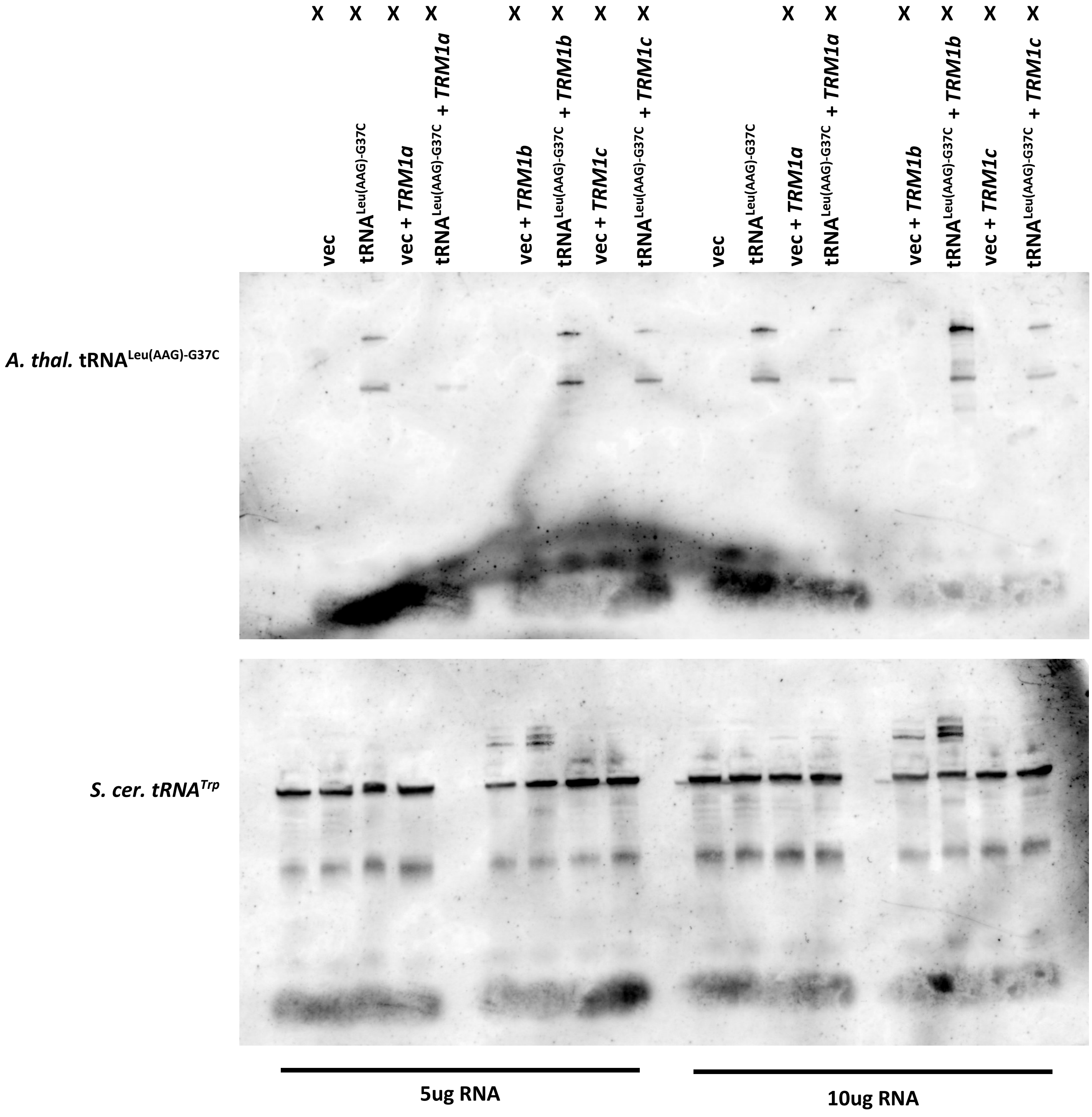

Fig. 3B. A. thaliana tRNA$^{Leu(AAG)}$-G37C is expressed in yeast cells.

RNA was extracted from indicated strains and analyzed by Northern blot. 5ug and 10ug of bulk RNA were analyzed by 10% PAGE and transferred to a nitrocellulose membrane. 5' Tye665-labeled oligonucleotide probes specific to the indicated tRNA were used for detection. The gel was visualized using a Typhoon 9200 scanner with a 620 BP30 Cy5 emission filter at high sensitivity. Brightness and contrast adjusted and applied equally to entire gel.

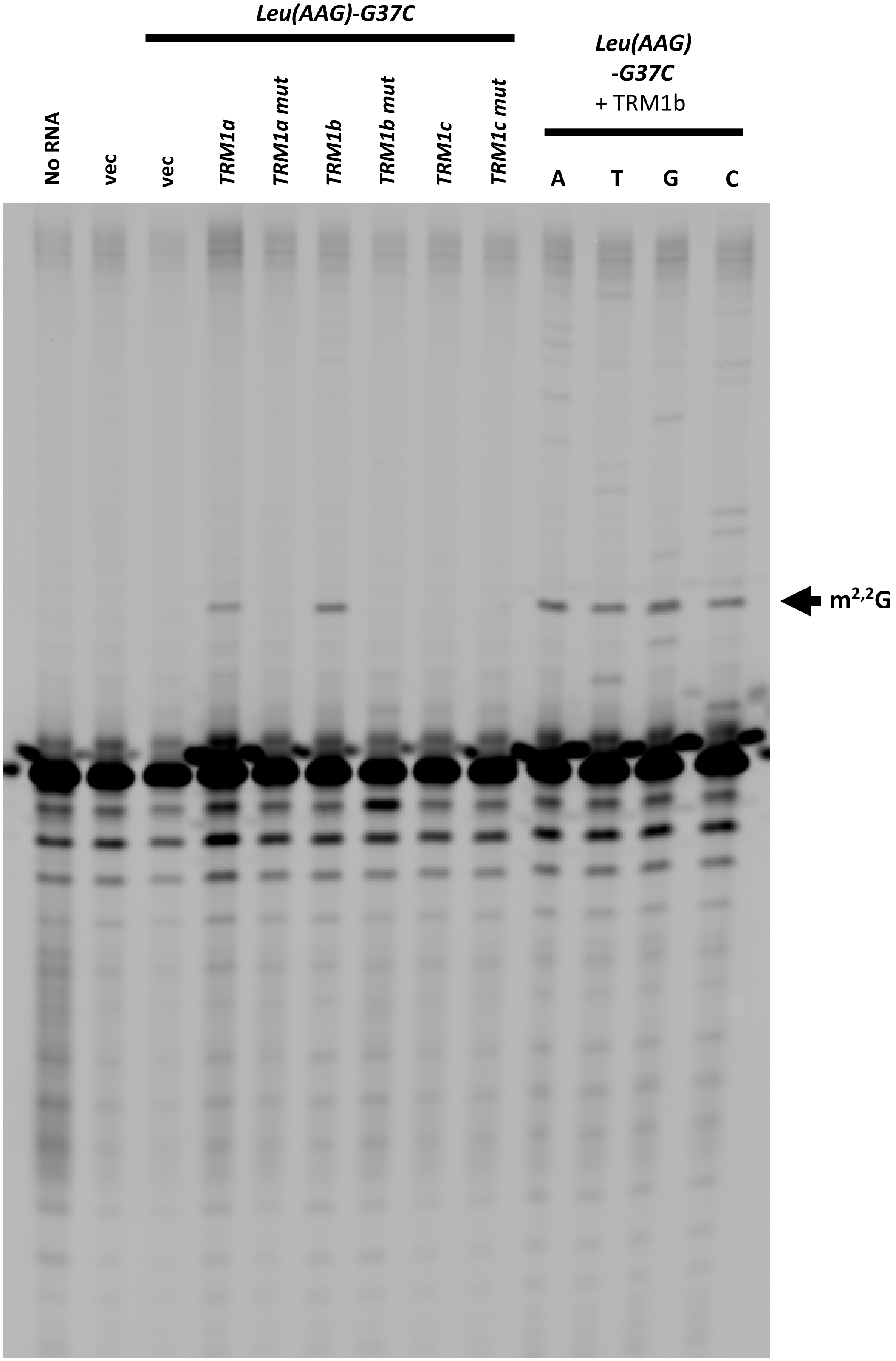

Fig. 3C. Identification of *TRM1* genes from *A. thaliana* in yeast cells.

TRM1a and TRM1b form $m^{2,2}G_{26}$ on *A. thaliana* tRNA. Bulk RNA was extracted from a *trm1Δ* yeast strain expressing the indicated plasmids and then analyzed by primer extension to *A. thaliana* $tRNA^{Leu(AAG)}$-G37C. 20 umol 5' Tye665-labeled oligonucleotides (Integrated DNA Technologies) was annealed to 6 µg RNA, heated to 95°C and slow cooled to 37°C. The entire reaction was then incubated with 1 mM dNTPs and 1.89 U of Avian Myeloblastosis Virus (AMV). Reactions were incubated at 37°C overnight, and analyzed by 15% PAGE with 7M Urea. The gel was placed between overhead projector sheets and visualized using a Typhoon 9200 scanner with a 620 BP30 Cy5 emission filter at high sensitivity.

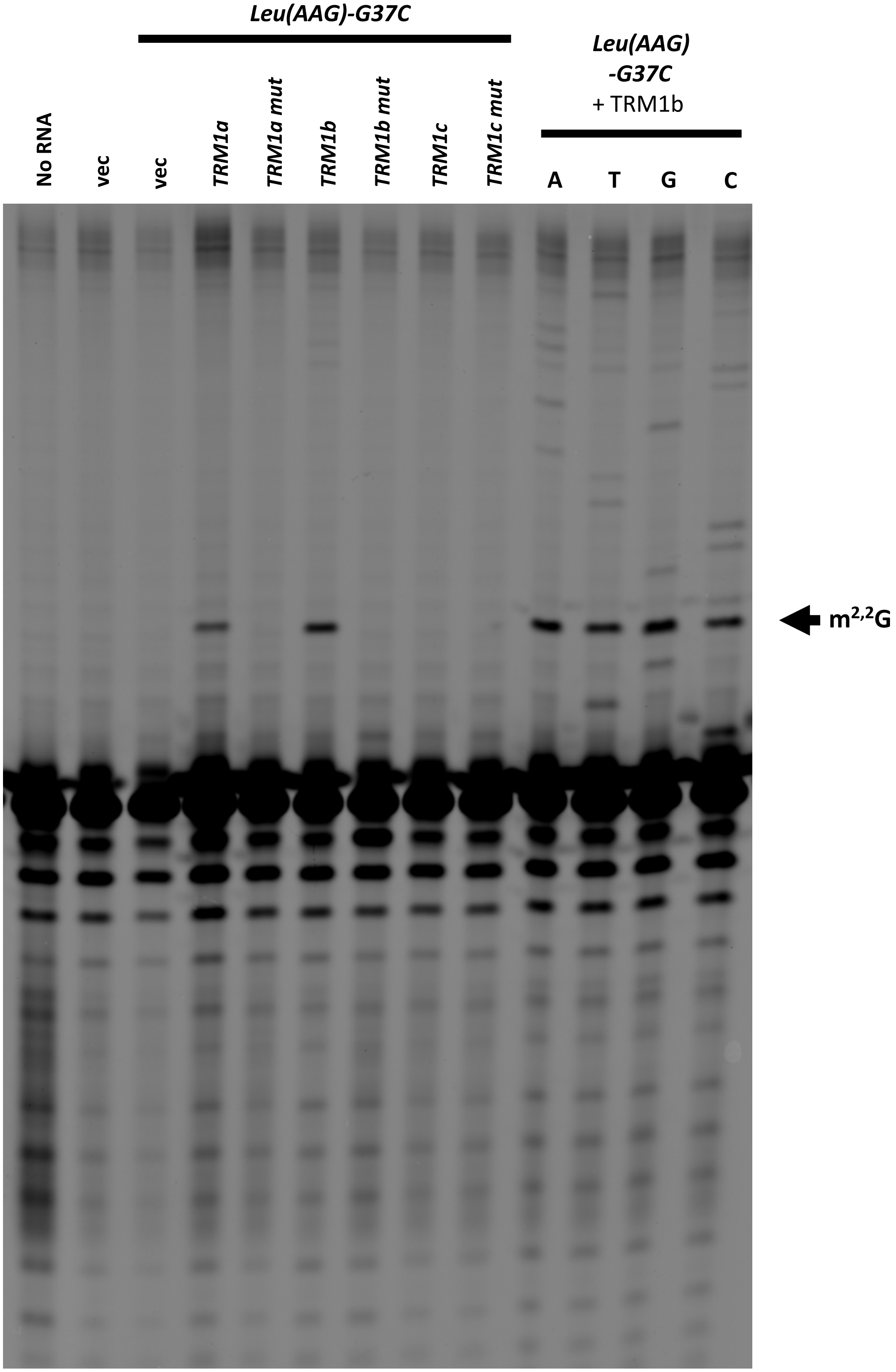

Fig. 3C. Identification of *TRM1* genes from *A. thaliana* in yeast cells.

TRM1a and TRM1b form $m^{2,2}G_{26}$ on *A. thaliana* tRNA. Bulk RNA was extracted from a *trm1Δ* yeast strain expressing the indicated plasmids and then analyzed by primer extension to *A. thaliana* $tRNA^{Leu(AAG)}$-G37C. 20 umol 5' Tye665-labeled oligonucleotides (Integrated DNA Technologies) was annealed to 6 µg RNA, heated to 95°C and slow cooled to 37°C. The entire reaction was then incubated with 1 mM dNTPs and 1.89 U of Avian Myeloblastosis Virus (AMV). Reactions were incubated at 37°C overnight, and analyzed by 15% PAGE with 7M Urea. The gel was placed between overhead projector sheets and visualized using a Typhoon 9200 scanner with a 620 BP30 Cy5 emission filter at high sensitivity. Brightness and contrast adjusted and applied equally to entire gel.

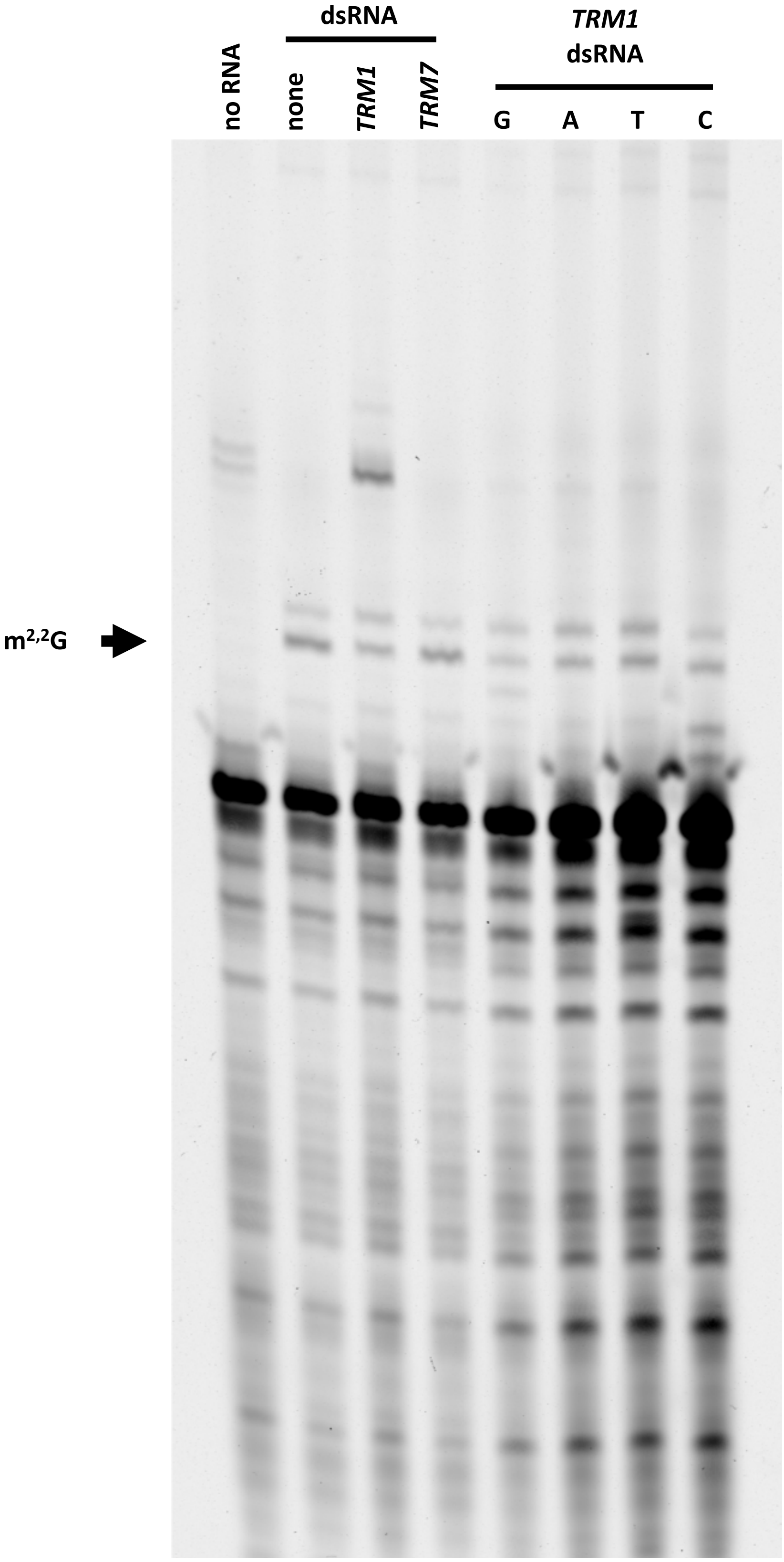

Fig. 4C. Knockdown of CG6388 by RNAi results in loss of a primer extension block consistent with $m^{2,2}G_{26}$.

S2R+ cells were treated twice over 6 days with 10 ug dsRNA to indicated gene. After harvest of cells, RNA was extracted and primer extension to $tRNA^{Tyr}$ was performed. 10 umol 5' Tye665-labeled oligonucleotides (Integrated DNA Technologies) was annealed to 8 µg RNA, heated to 95°C and slow cooled to 37°C. The entire reaction was then incubated with 1 mM dNTPs and 1.89 U of Avian Myeloblastosis Virus (AMV). Sequencing reactions were annealed similarly and extended overnight with the addition of 0.1mM ddNTP's. Reactions were incubated at 37°C overnight, and analyzed by 15% PAGE with 7M Urea. The gel was placed between overhead projector sheets and visualized using a Typhoon 9200 scanner with a 620 BP30 Cy5 emission filter at high sensitivity.

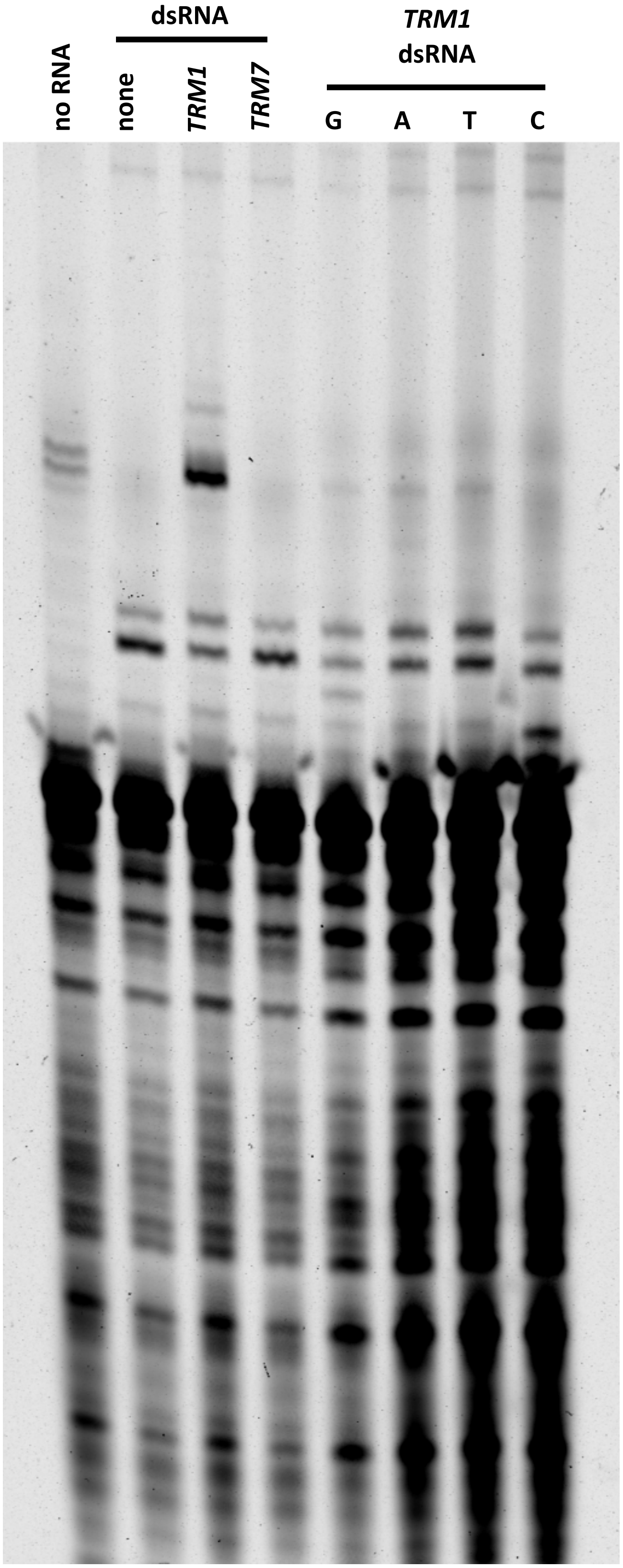

Fig. 4C. Knockdown of CG6388 by RNAi results in loss of a primer extension block consistent with $m^{2,2}G_{26}$.

S2R+ cells were treated twice over 6 days with 10 ug dsRNA to indicated gene. After harvest of cells, RNA was extracted and primer extension to $tRNA^{Tyr}$ was performed. 10 umol 5′ Tye665-labeled oligonucleotides (Integrated DNA Technologies) was annealed to 8 μg RNA, heated to 95°C and slow cooled to 37°C. The entire reaction was then incubated with 1 mM dNTPs and 1.89 U of Avian Myeloblastosis Virus (AMV). Sequencing reactions were annealed similarly and extended overnight with the addition of 0.1mM ddNTP's. Reactions were incubated at 37°C overnight, and analyzed by 15% PAGE with 7M Urea. The gel was placed between overhead projector sheets and visualized using a Typhoon 9200 scanner with a 620 BP30 Cy5 emission filter at high sensitivity. Brightness and contrast adjusted and applied equally to entire gel.

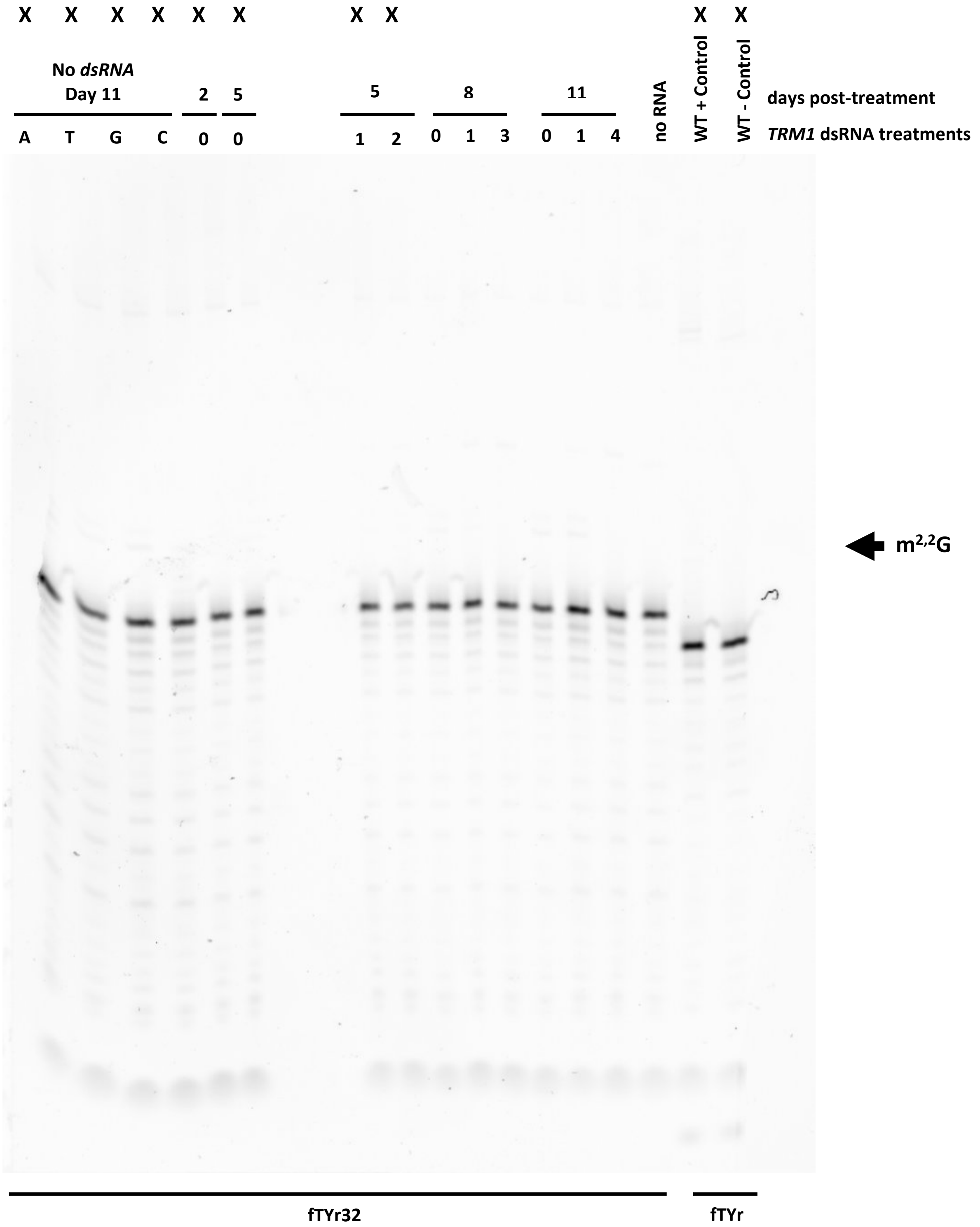

Fig. 4D. Time course of CG6388 knockdown by RNAi.

S2R+ cells were treated as indicated, RNA was extracted, and primer extension to tRNATyr was performed. 10 umol 5’ Tye665-labeled oligonucleotides (Integrated DNA Technologies) was annealed to 2 µg RNA, heated to 95°C and slow cooled to 37°C. The entire reaction was then incubated with 1 mM dNTPs and 1.89 U of Avian Myeloblastosis Virus (AMV). Sequencing reactions were annealed similarly and extended overnight with the addition of 0.1mM ddNTP’s. Reactions were incubated at 37°C overnight, and analyzed by 15% PAGE with 7M Urea. The gel was placed between overhead projector sheets and visualized using a Typhoon 9200 scanner with a 620 BP30 Cy5 emission filter at high sensitivity.

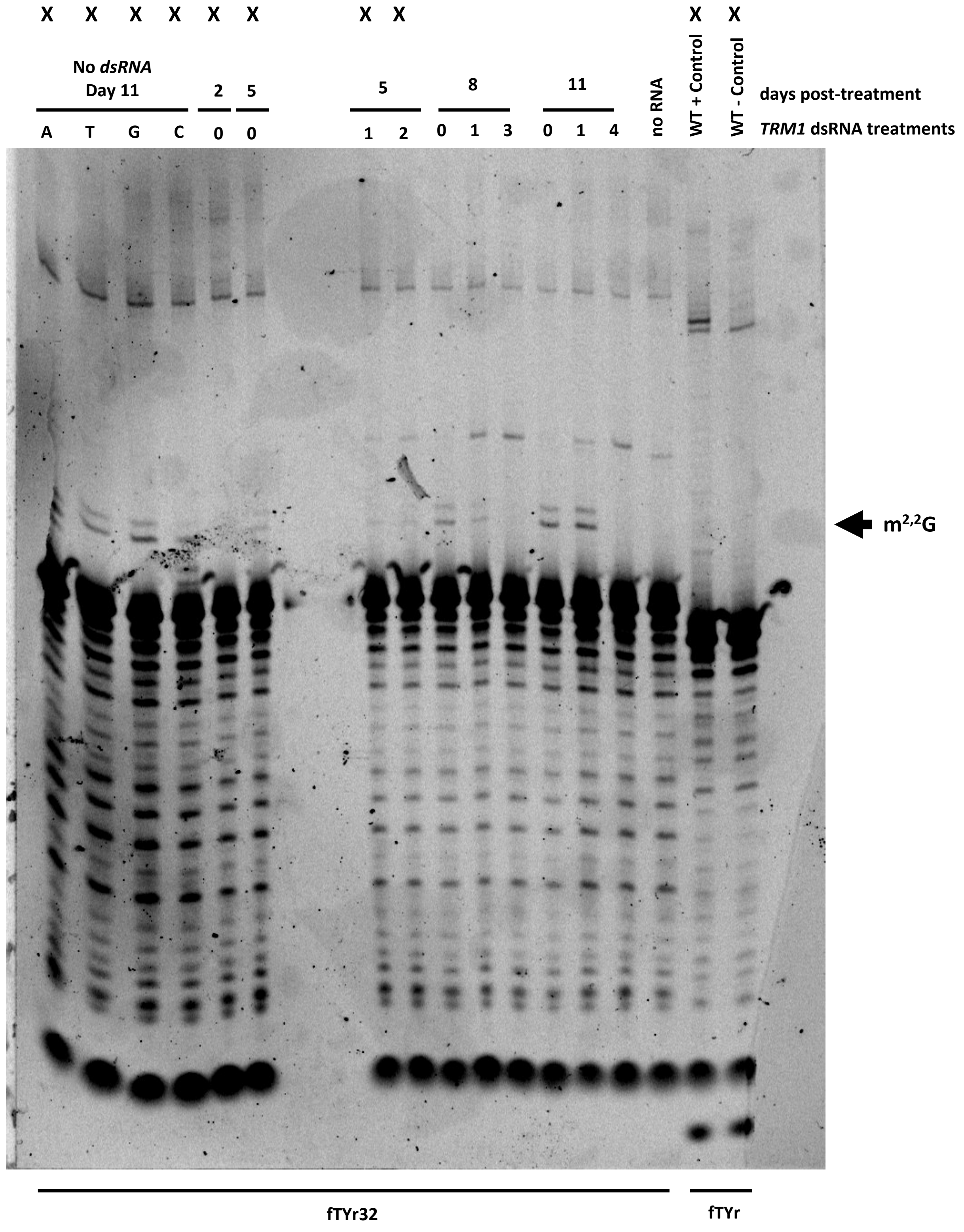

Fig. 4D. ***CG6388*** **encodes the TRM1 enzyme in** ***D. melanogaster*****.**

S2R+ cells were treated as indicated, RNA was extracted, and primer extension to tRNATyr was performed. 10 umol 5′ Tye665-labeled oligonucleotides (Integrated DNA Technologies) was annealed to 2 µg RNA, heated to 95°C and slow cooled to 37°C. The entire reaction was then incubated with 1 mM dNTPs and 1.89 U of Avian Myeloblastosis Virus (AMV). Sequencing reactions were annealed similarly and extended overnight with the addition of 0.1mM ddNTP's. Reactions were incubated at 37°C overnight, and analyzed by 15% PAGE with 7M Urea. The gel was placed between overhead projector sheets and visualized using a Typhoon 9200 scanner with a 620 BP30 Cy5 emission filter at high sensitivity. Brightness and contrast adjusted and applied equally to entire gel.

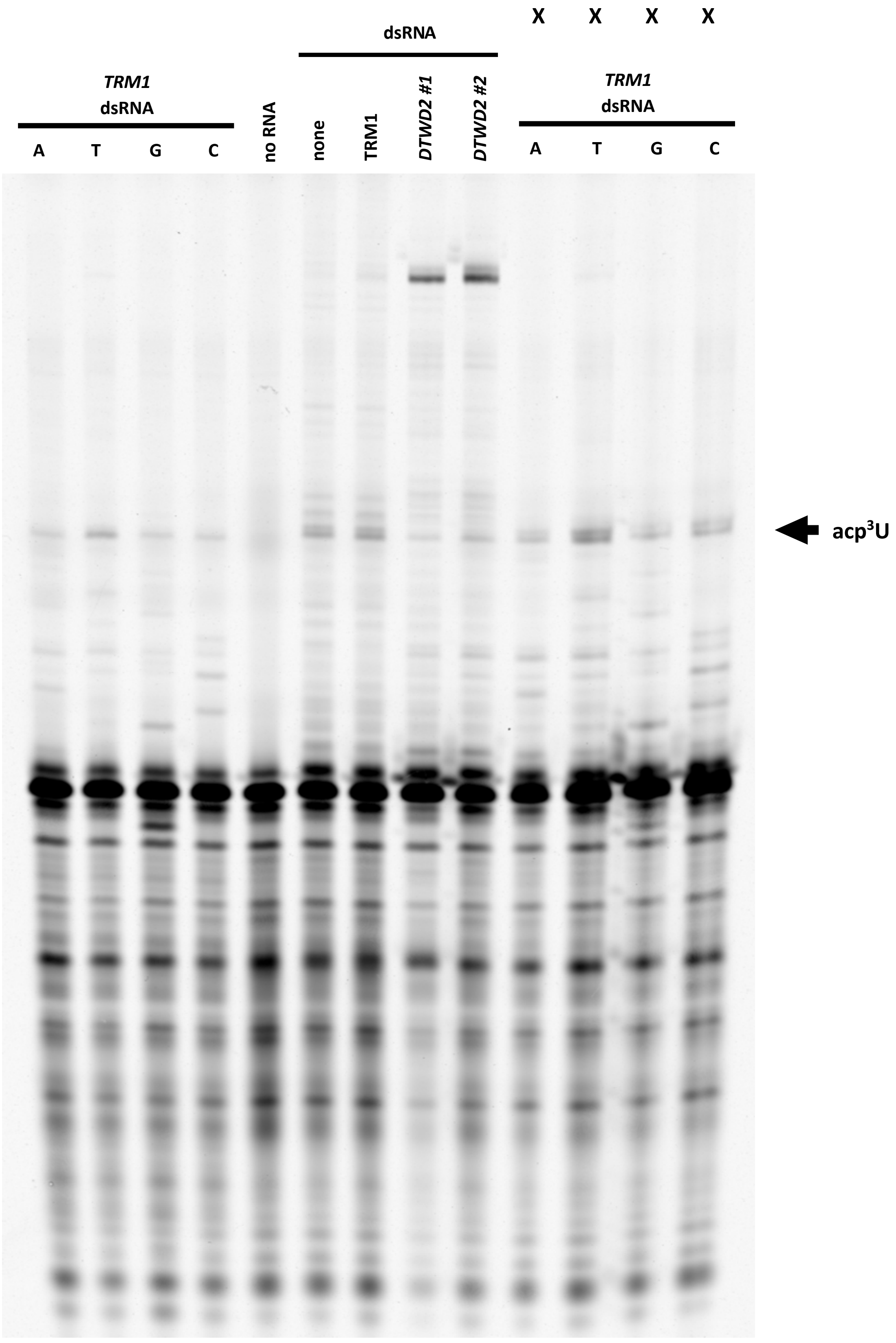

Fig. 5C. Knockdown of CG10050 by RNAi results in loss of a primer extension block consistent with acp^{3}U$_{20b}$ on tRNA.

S2R+ cells were treated three times over 8 days with 10 ug dsRNA to indicated gene. After harvest of cells, RNA was extracted and primer extension to tRNAVal(CAC) was performed. 20 umol 5' Tye665-labeled oligonucleotides (Integrated DNA Technologies) was annealed to 10 µg RNA, heated to 95°C and slow cooled to 37°C. The entire reaction was then incubated with 1 mM dNTPs and 1.89 U of Avian Myeloblastosis Virus (AMV). Sequencing reactions were annealed similarly and extended overnight with the addition of 0.1 mM ddNTP's. Reactions were incubated at 37°C overnight, and analyzed by 15% PAGE with 7M Urea. The gel was placed between overhead projector sheets and visualized using a Typhoon 9200 scanner with a 620 BP30 Cy5 emission filter at high sensitivity.

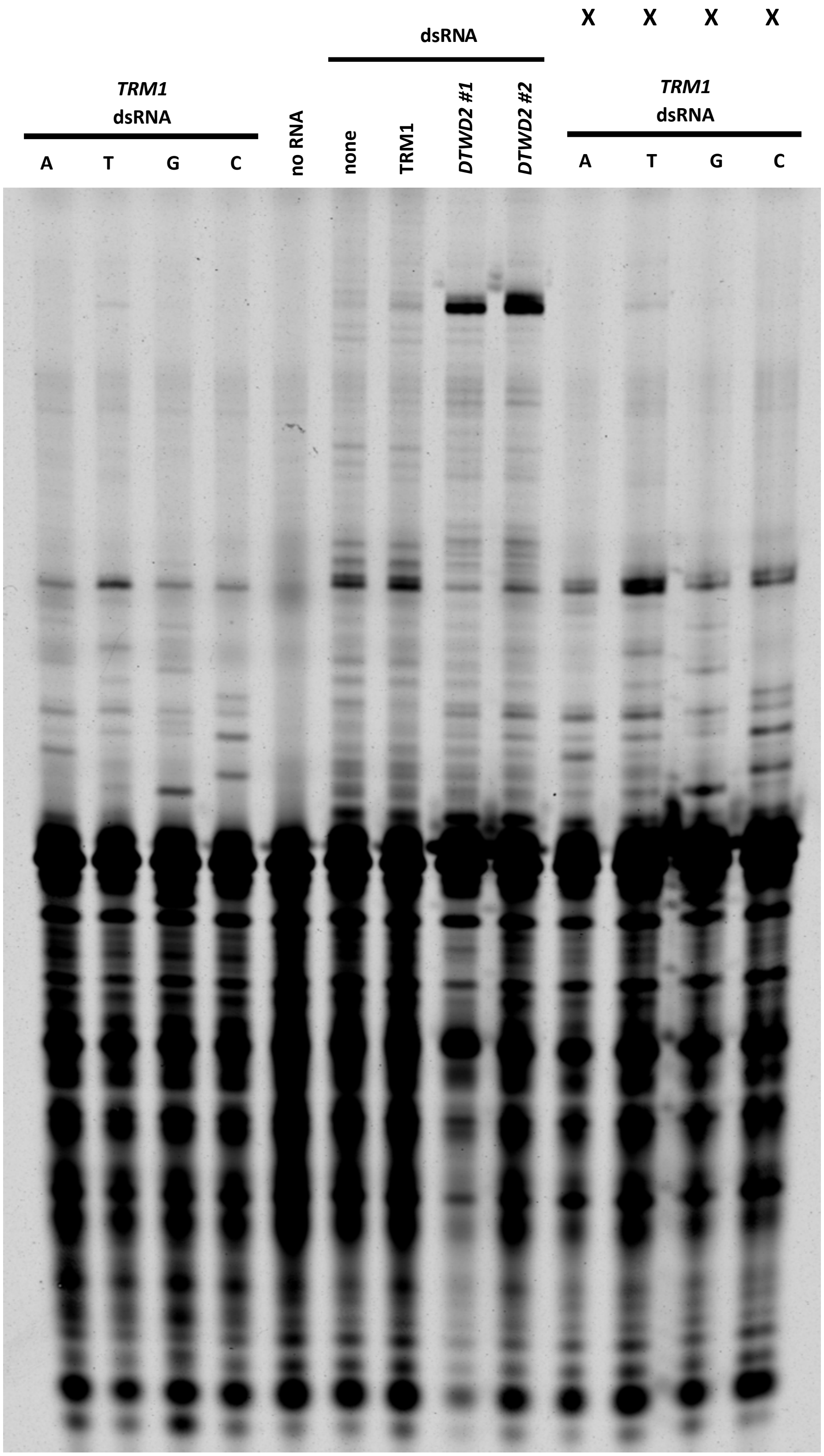

Fig. 5C. Knockdown of CG10050 by RNAi results in loss of a primer extension block consistent with acp^{3}U$_{20b}$ on tRNA.

S2R+ cells were treated three times over 8 days with 10 ug dsRNA to indicated gene. After harvest of cells, RNA was extracted and primer extension to tRNAVal(CAC) was performed. 20 umol 5' Tye665-labeled oligonucleotides (Integrated DNA Technologies) was annealed to 10 µg RNA, heated to 95°C and slow cooled to 37°C. The entire reaction was then incubated with 1 mM dNTPs and 1.89 U of Avian Myeloblastosis Virus (AMV). Sequencing reactions were annealed similarly and extended overnight with the addition of 0.1 mM ddNTP's. Reactions were incubated at 37°C overnight, and analyzed by 15% PAGE with 7M Urea. The gel was placed between overhead projector sheets and visualized using a Typhoon 9200 scanner with a 620 BP30 Cy5 emission filter at high sensitivity. Brightness and contrast adjusted and applied equally to entire gel.

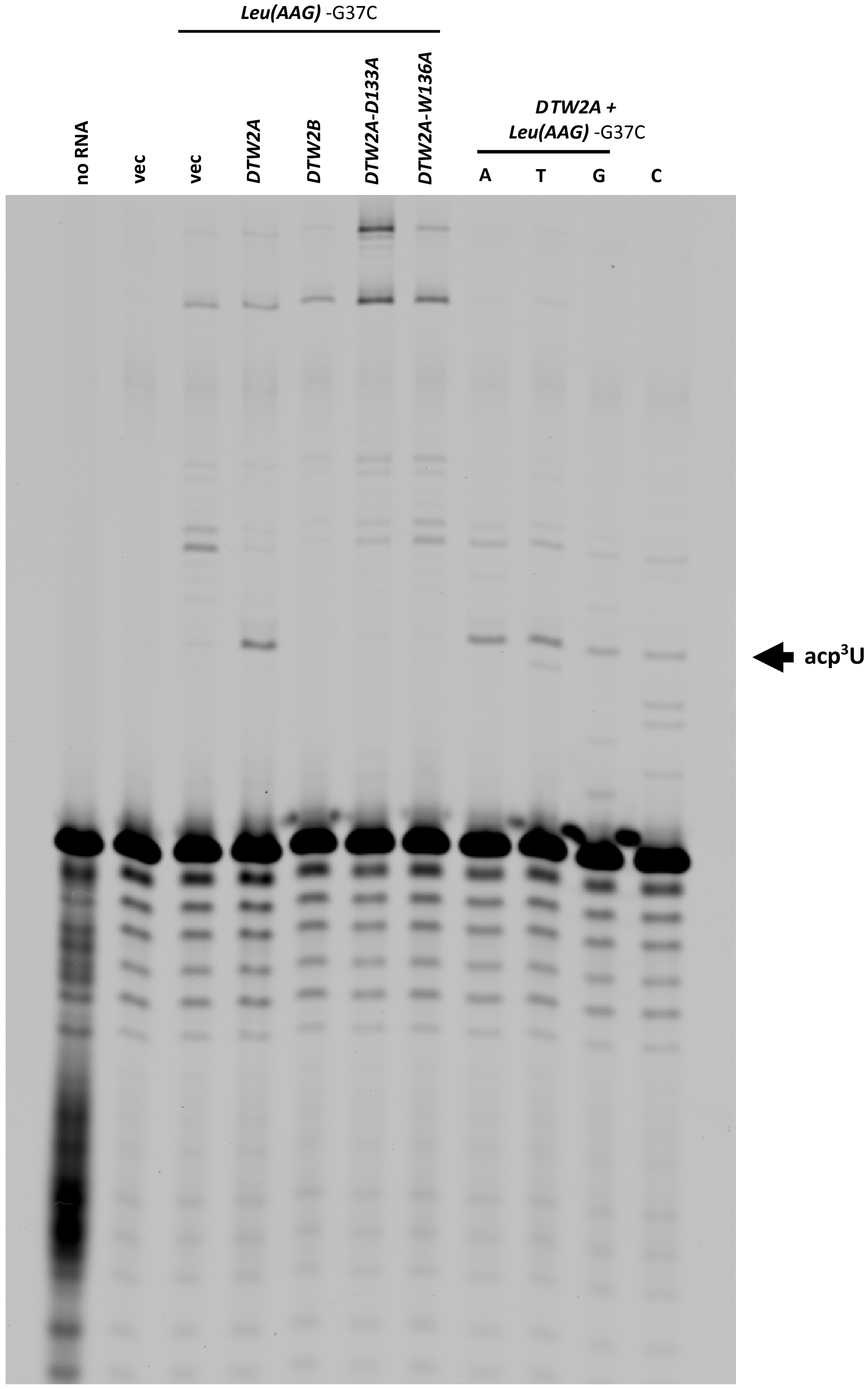

Fig. 6B. DTW2A expression results in a primer extension block consistent with acp3U20b and the D133 and W136 residues of DTWD2A are required for modification activity on A. thaliana tRNA.

Bulk RNA was extracted from a trm1Δ yeast strain expressing the indicated plasmids and then analyzed by primer extension to A. thaliana tRNALeu(AAG)-G37C. 20 umol 5′ Tye665-labeled oligonucleotides (Integrated DN A Technologies) was annealed to 6 µg RNA, heated to 95°C and slow cooled to 37°C. The entire reaction was then incubated with 1 mM dNTPs and 1.89 U of Avian Myeloblastosis Virus (AMV). Sequencing reactions were annealed sim ilarly and extended overnight with the addition of 0.1mM ddNTP's. Reactions were incubated at 37°C overnight, and analyzed by 15% PAGE with 7M Urea. The gel was placed between overhead projector sheets and visualized using a Typhoon 9200 scanner with a 620 BP30 Cy5 emission filter at high sensitivity.

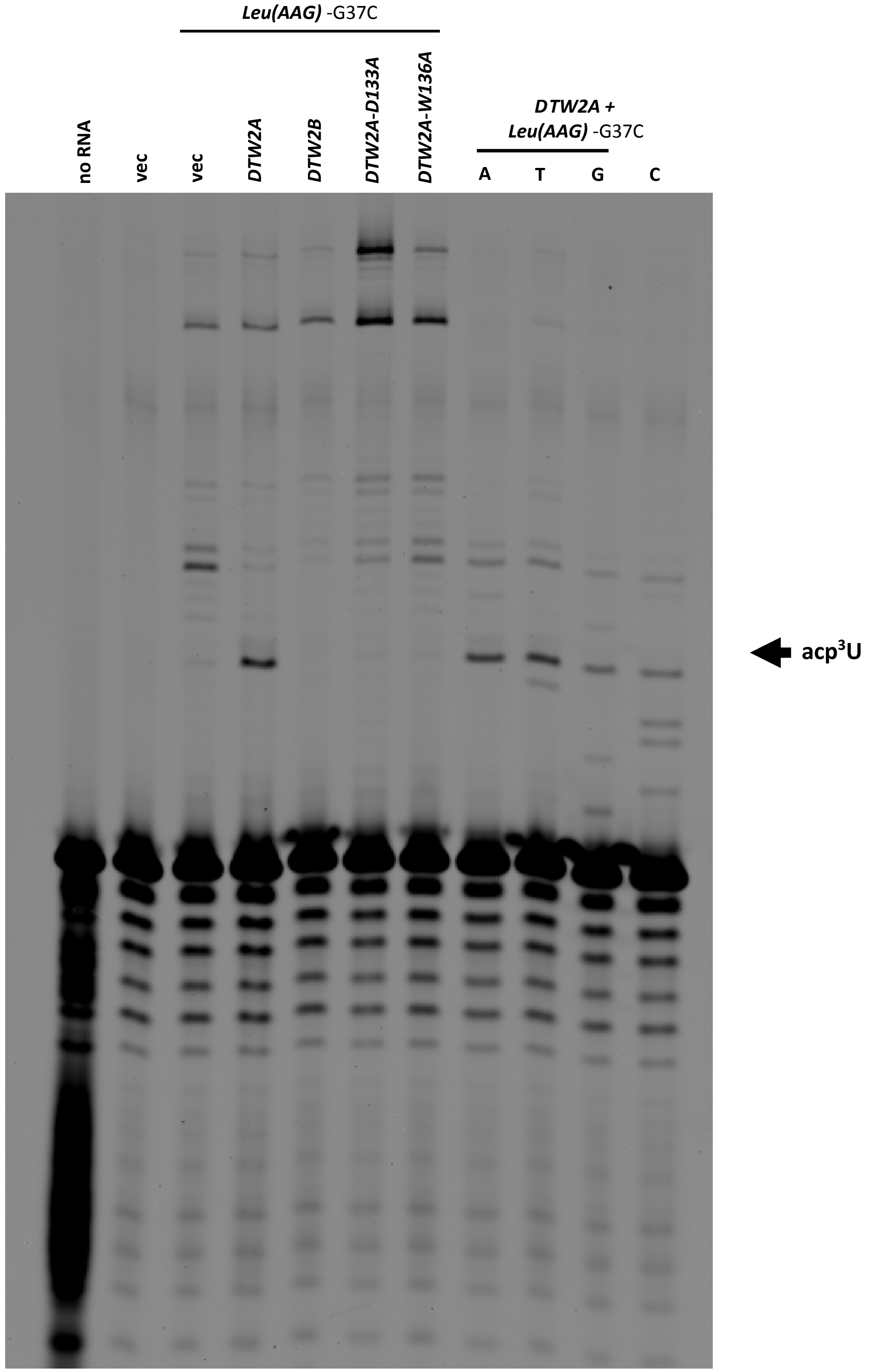

Fig. 6B. DTW2A expression results in a primer extension block consistent with acp3U20b and the D133 and W136 residues of DTWD2A are required for modification activity on A. thaliana tRNA.

Bulk RNA was extracted from a trm1Δ yeast strain expressing the indicated plasmids and then analyzed by primer extension to A. thaliana tRNALeu(AAG)-G37C. 20 umol 5′ Tye665-labeled oligonucleotides (Integrated DN A Technologies) was annealed to 6 µg RNA, heated to 95°C and slow cooled to 37°C. The entire reaction was then incubated with 1 mM dNTPs and 1.89 U of Avian Myeloblastosis Virus (AMV). Sequencing reactions were annealed sim ilarly and extended overnight with the addition of 0.1mM ddNTP's. Reactions were incubated at 37°C overnight, and analyzed by 15% PAGE with 7M Urea. The gel was placed between overhead projector sheets and visualized using a Typhoon 9200 scanner with a 620 BP30 Cy5 emission filter at high sensitivity. Brightness and contrast adjusted and applied equally to entire gel.
